# Digital tools for direct assessment of autism risk during early childhood: A systematic review

**DOI:** 10.1177/13623613221133176

**Published:** 2022-11-07

**Authors:** Debarati Mukherjee, Supriya Bhavnani, Georgia Lockwood Estrin, Vaisnavi Rao, Jayashree Dasgupta, Hiba Irfan, Bhismadev Chakrabarti, Vikram Patel, Matthew K Belmonte

**Affiliations:** 1 Indian Institute of Public Health – Bengaluru, Public Health Foundation of India, India; 2 Child Development Group, Sangath, India; 3 Birkbeck, University of London, UK; 4 University of East London, UK; 5 Institute for Democracy and Economic Affairs (IDEAS), Malaysia; 6 University of Reading, UK; 7 Ashoka University, India; 8 India Autism Center, India; 9 Harvard Medical School, USA; 10 Harvard T.H. Chan School of Public Health, USA; 11 The Com DEALL Trust, India; 12 Nottingham Trent University, UK

**Keywords:** ASD, assessments, computer, digital, gamified, low-resource, mHealth, scalable, smartphone, tablet, virtual reality

## Abstract

Current challenges in early identification of autism spectrum disorder lead to significant delays in starting interventions, thereby compromising outcomes. Digital tools can potentially address this barrier as they are accessible, can measure autism-relevant phenotypes and can be administered in children’s natural environments by non-specialists. The purpose of this systematic review is to identify and characterise potentially scalable digital tools for direct assessment of autism spectrum disorder risk in early childhood. In total, 51,953 titles, 6884 abstracts and 567 full-text articles from four databases were screened using predefined criteria. Of these, 38 met inclusion criteria. Tasks are presented on both portable and non-portable technologies, typically by researchers in laboratory or clinic settings. Gamified tasks, virtual-reality platforms and automated analysis of video or audio recordings of children’s behaviours and speech are used to assess autism spectrum disorder risk. Tasks tapping social communication/interaction and motor domains most reliably discriminate between autism spectrum disorder and typically developing groups. Digital tools employing objective data collection and analysis methods hold immense potential for early identification of autism spectrum disorder risk. Next steps should be to further validate these tools, evaluate their generalisability outside laboratory or clinic settings, and standardise derived measures across tasks. Furthermore, stakeholders from underserved communities should be involved in the research and development process.

**Lay abstract:**

The challenge of finding autistic children, and finding them early enough to make a difference for them and their families, becomes all the greater in parts of the world where human and material resources are in short supply. Poverty of resources delays interventions, translating into a poverty of outcomes. Digital tools carry potential to lessen this delay because they can be administered by non-specialists in children’s homes, schools or other everyday environments, they can measure a wide range of autistic behaviours objectively and they can automate analysis without requiring an expert in computers or statistics. This literature review aimed to identify and describe digital tools for screening children who may be at risk for autism. These tools are predominantly at the ‘proof-of-concept’ stage. Both portable (laptops, mobile phones, smart toys) and fixed (desktop computers, virtual-reality platforms) technologies are used to present computerised games, or to record children’s behaviours or speech. Computerised analysis of children’s interactions with these technologies differentiates children with and without autism, with promising results. Tasks assessing social responses and hand and body movements are the most reliable in distinguishing autistic from typically developing children. Such digital tools hold immense potential for early identification of autism spectrum disorder risk at a large scale. Next steps should be to further validate these tools and to evaluate their applicability in a variety of settings. Crucially, stakeholders from underserved communities globally must be involved in this research, lest it fail to capture the issues that these stakeholders are facing.

## Introduction

Autism spectrum disorder (ASD), which affects 1 in 132 people globally with little regional variation (Baxter et al., 2015), is characterised by persistent difficulties in social communication and behavioural flexibility (American Psychiatric Association, 2013). ASD is often comorbid with epilepsy, gastrointestinal disorders, sleep disorders and other neurodevelopmental disorders or conditions, such as intellectual disability and attention-deficit hyperactivity disorder (Doshi-Velez et al., 2014; Levy et al., 2010). Fine and gross motor atypicalities and sensory sensitivity are also commonly observed in individuals with ASD (American Psychiatric Association, 2013).

Early childhood, as a period of rapid brain development, presents great opportunity and risk in shaping the developmental potential of all children, including those with neurodevelopmental disorders, such as ASD (Black et al., 2017). Early detection of ASD and intervention when the brain is most plastic lead to the best outcomes (Estes et al., 2015; Flanagan et al., 2012; Kasari et al., 2012). However, current challenges in diagnosing ASD in low-resource settings lead to significant delays in detection, and therefore in triaging to appropriate interventions (Mukherjee et al., 2014; Patra et al., 2020). For example, inadequate parental and community awareness about the red flags of autism compromise help-seeking behaviours (Divan et al., 2021). The available diagnostic tools demand administration by skilled and trained specialists, a scarce resource in most settings (Durkin et al., 2015). Moreover, these specialists are concentrated in urban areas or expensive private clinics inaccessible to the large majority of the population. Standardised assessment methods for ASD are lengthy, proprietary, globally priced and therefore not feasible for large-scale deployment (Durkin et al., 2015). However, the more scalable autism screening measures that depend on parent-report questionnaires are often unreliable, as they assume parental knowledge about autism symptoms is often lacking in communities with low maternal education and limited awareness about child development (Dawson & Sapiro, 2019; Khowaja et al., 2015). All these factors contribute to a failure in timely identification of children with autism, resulting in a large ‘detection gap’ (Dasgupta et al., 2016), with consequent delays in receiving a diagnosis and being placed on appropriate care pathways (Bhavnani et al., 2022).

Therefore, there is a critical need to develop scalable tools for autism risk assessment in the early years to leverage into improved outcomes throughout the life course. Digital tools have tremendous potential to address the scalability issue as portable computers and smart devices are now highly accessible across the globe, even in low-resource settings (Istepanian & AlAnzi, 2020). Over 5 billion people, representing more than two-thirds of the global population, have access to smart phones (World Health Organization Global Observatory for eHealth, 2011). The potential for these mHealth tools to be administered in children’s natural environments, such as homes and schools (Sapiro et al., 2019), and reports generated through automated analysis of objective and high-resolution dimensional data make them feasible for administration by non-specialist providers, including parents. This natural environmental setting also garners more representative behavioural observations. By leveraging the multitude of sensors, such as cameras, audio recorders and touch-sensitive displays, digital tools can measure a wide range of autism-relevant phenotypes, including differences in social-emotional, motor and language skills, helping to capture the heterogeneity of the autism phenotype and providing a comprehensive view of the child’s strengths and weaknesses. Alongside clinical practice, this potential for task-sharing for ASD risk screening (Naslund et al., 2019) protects the time and efforts of highly skilled specialists towards diagnosis and treatment of the small fraction who screen positive. Finally, direct assessment of child behaviour through performance-based tasks picks up quantitative information complementary to parent reports that depend on awareness about autism-related behaviours (Dawson & Sapiro, 2019).

Recent reviews have summarised the evidence on the use of digital tools for autism assessment based on parent-report questionnaires (Marlow et al., 2019; Stewart & Lee, 2017), and the more technologically challenging eye-tracking (Alcañiz et al., 2022; Mastergeorge et al., 2021; Papagiannopoulou et al., 2014), electroencephalography (O’Reilly et al., 2017) and magnetic resonance imaging (Sato & Uono, 2019) methods. However, these tools are not ideal for screening in low-resource settings either because of their dependence on parent reports which may be unreliable, the requirement for expensive equipment and software typically administered in controlled laboratories or the need for high levels of manual input and expertise in analysing the data. In contrast, digital tasks administered using more accessible and portable devices, such as computers, tablets and smartphones, and amenable to automated analysis of child responses and behaviours, have a much greater potential to scale since they are suitable for task-sharing approaches (Naslund et al., 2019). However, a comprehensive review of the characteristics and utility of scalable digital tools for direct assessment of autism risk during early childhood is critically missing. This omission is especially significant in terms of their potential to be further developed into valid screening tools deployable at scale in low-resource settings.

This review attempts to bridge this gap by addressing the following questions:

1. What types of digital tasks are being used for direct assessment of autism risk during early childhood, and which diagnostic (DSM-5) criteria and specific ASD-related phenotype do they target?
2. How well are these tools (and specific metrics derived therefrom) able to discriminate between ASD and typically developing (TD) groups in case–control studies?
3. What are the implementation strategies of these tools in relation to hardware and configuration, passive or active task, personnel and time taken for administration?

## Methods

### Search

While this review focuses on scalable digital tools to assess autism risk during early childhood (0–8 years), it is based on a subset of papers identified from a more comprehensive search of peer-reviewed articles describing scalable digital tools for assessment of autism and attention deficit hyperactivity disorder across 0–18 years. Four databases (PubMed, PsycInfo, Scopus and Web of Science) were searched in two phases to retrieve relevant articles. During the first phase conducted in May 2018, no date restrictions were applied. The second phase, specific to this review topic, updated the original search by including relevant articles published from June 2018 through October 2020. Specific keywords used for Phases 1 and 2 are presented in Supplementary Table 1.

### Study selection and data extraction

Search results from selected databases were imported into the Rayyan software (https://rayyan.ai/) (Ouzzani et al., 2016). Titles and abstracts of the imported articles were screened by three reviewers (D.M., V.R. and J.D.) during Phase 1 and two reviewers during Phase 2 (D.M. and G.L.E.) using the inclusion/exclusion criteria described below. Screening results were ‘unblinded’ for group review weekly, and conflicts were resolved through group consensus. Full texts of included articles were downloaded and screened for eligibility. Data were extracted from included articles.

### Eligibility criteria

Scalable digital tools were defined as those that collected and analysed data in a digital format using desktop or mobile devices (laptop, tablets, smartphones or any other mobile smart device). Included studies either required the child to engage actively with tasks presented on the device or used the device to acquire data from the child passively (e.g. via voice or video recording).

The inclusion criteria were (a) peer-reviewed primary research articles published in the English language; (b) case–control study design with at least two groups – ASD and TD comparison group (papers with additional atypical comparison groups, such as neurodevelopmental disorders other than ASD, were included) and (c) mean age of the participant groups ⩽ 8 years (defined as early childhood by the World Health Organization, 2020). The exclusion criteria were (a) digital tools that collected only parent-report data since this review focused on digital tools for *direct* child assessment; (b) tools that required manual coding of child behaviour post data collection since this method is time-consuming and subjective, therefore unlikely to scale in low-resource settings with limited numbers of trained specialists and (c) studies that only reported the acceptability and feasibility testing of the tool or used a small sample (*N* < 5 per group) since one of the primary objectives of this review was to evaluate the discriminative ability of these novel tools for early identification of ASD risk.

### Analysis

For each included study, data were tabulated to describe the task(s) presented to the child, the experimental setup, device(s) used and the format in which the child’s response was recorded. The primary metric(s) used to determine group differences also were tabulated, along with the main findings (Table 1). A brief description of the participants (mean and standard deviation or range of the age distribution, sample size and gender distribution) was included in the table. Papers were grouped based on the *Diagnostic and Statistical Manual of Mental Disorders, Fifth Edition* (DSM-5) diagnostic criteria they covered, along with a mention of the specific developmental domain/phenotype assessed and the country in which the research was based. A more detailed description of the study groups and methods is presented in Supplementary Table 2 (demographic details, inclusion/exclusion criteria, standardised tools used to diagnose children with autism, level of functioning of the participants and sample size of children recruited versus completing the tasks, along with reasons for loss to follow-up).

**Table 1.** Characteristics and discriminating ability of scalable digital tools to assess ASD risk during early childhood.

Citation, specific measure	Participant details^a^ (Mean age, sample size, gender distribution)	Device specifications	Experimental setup	Primary metric (s) and summary results
DSM-5 criteria: Social communication/Social interaction				
Anzulewicz et al. (2016) Specific measure: ‘Sharing’ and fine-motor drawing movements Country: United Kingdom	1. ASD group^a^ *M*_age_ (*SD*) = 53 (11) months, *N* = 35, %male = 67.57 2. TD group *M*_age_ (*SD*) = 55 (11) months, *N* = 45, %male = 71.11	Tablet computer (iPad mini) running standard iOS version 7.0	Task description: Two commercially available computerised games. Child response: Game 1 (Sharing): Slice a piece of food by tapping on it and distributing it evenly among four characters. Characters expressed positive/negative emotions based on equal/unequal distribution of food. Game 2 (Creativity): Trace a picture followed by colouring using finger motions. #trials: 1 Setting: Not specified Duration: 5 min	In total, 262 features from touch data (gestures on screen) and tablet’s inertial movement sensors acquired. Machine learning (ML) using touch and sensor data to predict child’s diagnostic group (TD vs ASD). Significant differences in the following features: 1. Impact Force and Gesture Pressure: ASD > TD 2. Distribution of forces into the device: (gyroscope data) patterns of force distribution different 3. Mean gesture velocity: ASD > TD 4. Mean area occupied by a gesture: ASD > TD 5. Gestures in distal parts of the screen: ASD > TD 6. Minimum duration of a screen tap: ASD < TD 7. ML prediction accuracy: Max AUC = 0.93, sensitivity = 0.83, specificity = 0.85
Ruta et al. (2017) Specific measure: Social preference Country: Italy	1. ASD group *M*_age_ (*SD*) = 39.9 (11.5) months, *N* = 21, %male = 85.7 2. TD group *M*_age_ (*SD*) = 45.5 (10.7) months, *N* = 37, %male = 48.6	Tablet computer (iPad)	Task description: Deliberate choice task with two pictures – non-social (toy-train) and social (smiling face) hidden under buttons. Button position was randomised in each trial. Control trials comprised scrambled images of the same pictures. Child response: Tap on button to show picture of choice. #trials: 8 per condition Setting: Laboratory/Home Duration: 5 min	1. Proportion of button taps to access social image: Significant difference (ASD < TD) No difference in control trials (scrambled images)
Chetcuti et al. (2019) Specific measure: Social imitation of simple versus complex motor tasks Country: Australia	1. ASD group *M*_age_ (*SD*) = 41.77 (8.5) months, *N* = 35, %male = 80 2. TD group *M*_age_ (*SD*) = 44.7 (13.64) months, *N* = 20, %male = 65	Tablet computer (iPad) with iPad application Slide & Spin	Task description: Four on-screen targets presented on the screen, manipulable using tap, drag, swipe and rotate actions. Child response: Imitate five-action sequence under four conditions (2 social × 2 motor complexity). Social comprised a socially responsive versus aloof instructor. Motor complexity involved low (five consecutive taps) versus high (multiple motor actions) complexity sequences. #trials: 1 per condition Setting: Laboratory/Home Duration: Not specified	1. Number of correct imitations by condition: Significant difference in high-motor demand task (ASD < TD). No difference in low-motor demand task. No difference based on social condition.
Carlsson et al. (2018) Specific measure: False-belief understanding Country: Sweden	1. ASD group^a^ *M*_age_ (*SD*) = 91.19 (10.8) months, *N* = 52, %male = 79.41 2. TD group *M*_age_ (*SD*) = 89.99 (10.8) months, *N* = 98, %male = 51.02	Tablet computer (specifications not provided)	Task description: Watch a short film adapted from Sally–Anne task. Facilitating effect of language support assessed using three auditory conditions: (1) narrative; (2) silent and (3) interference. Child response: Respond to questions by tapping on one of two yellow circles (correct and incorrect ROI). Data saved in the device. #trials: 2 per condition Setting: Clinic (ASD); Quiet school room (TD) Duration: 3–4 min	1. Task completion: 100% in TD group, 75% in ASD group 2. Accuracy (two successful trials per condition): Significant difference (ASD < TD) in narrative and silent conditions; same trend (but not significant) in interference condition
Jones et al. (2018) Specific measure: Statistical learning Country: USA	1. ASD group^a^ *M*_age_ (*SD*) = 64.67 (16.07) months, *N* = 56, %male = 80.36 2. TD group *M*_age_ (*SD*) = 60.11 (16.19) months, *N* = 68, %male = 55.88	Tablet computer (iPad)	Task description: Statistical learning task: sequence of two images (cue followed by target/distractor) presented for 2 s each. Cues were either high frequency (HF) or low frequency (LF), indicating likeliness of the next image to be a target or distractor. Child response: Tap on the target image, avoid distractor image. #trials: 7 runs of 24 trials each; 84 trials preceded by LF cue and HF cue, respectively. Setting: Laboratory Duration: Not specified	1. Accuracy: No difference. 2. Reaction time: Significant difference (ASD > TD). Unique RT patterns in TD (quadratic pattern in LF but not HF) versus ASD (linear pattern in both conditions). 3. Bayes classification to determine degree to which ASD child’s RT pattern was similar to TD group: ASD children with less severe autism symptoms (Social Responsiveness Scale-2) had similar learning profile to TD group.
Campbell et al. (2019) Specific measure: Social attention and response to name Country: USA	1. ASD group *M*_age_ (*SD*) = 26.19 (4.07) months, *N* = 22, %male = 77.27 2. TD group *M*_age_ (*SD*) = 21.91 (3.78) months, *N* = 82, %male = 58.54. Includes eight children with a diagnosis of language or developmental delay sufficient to qualify for speech or developmental therapy	Tablet computer (iPad)	Task description: Set of developmentally appropriate videos presented on the screen (cascading bubbles, mechanical bunny, animal puppets interacting with each other). During three of the videos, ‘Call Name’ appeared on the screen. The examiner, standing behind the child, called the child’s name loudly. Child response: The front camera on the tablet recorded the child’s video in response to name being called. ML algorithm automatically tracked head position. #trials: 3 prompts to call child’s name Setting: Laboratory Duration: 5 min	1. Task engagement (time looking at videos or people in the room): Significant difference (ASD < TD) 2. Orienting to name: a. At least once across three trials: No difference b. Multiple times across three trials: Significant difference (ASD < TD) c. Latency to orient to name: Significant difference (ASD > TD)
Gale et al. (2019) Specific measure: Social preference Country: Norway	1. ASD group *M*_age_ (*SD*) = 59.3 (18.3) months, *N* = 27, %male = 77.8 2. TD group *M*_age_ (*SD*) = 34.5 (12.3) months, *N* = 40, %male = 55	Tablet computer (Samsung Galaxy)	Task description: Study 1 and 2: Blurred videos of social (faces of people and dogs) and non-social (abstract moving geometric patterns) stimuli presented side-by-side. Study 3: Any one stimulus presented to assess reinforcement strength. Child response: Study 1 and 2: Tap on (blurred) video of choice. Chosen video grew larger and became clearly visible for 2 s. Study 3: Tap on stimuli (social or non-social) multiple times (progressively increasing – 2, 4, 6 times across trials – to view video clearly). #trials: 8 sessions Setting: child’s home, nursery or clinic Duration: 90 s for each session	1. Study 1 and 2: Proportion of taps on social videos: Significant difference (ASD < TD) 2. Study 3 (reinforcement strength): a. Proportion of taps to access non-social stimuli: Significant difference (ASD > TD). b. Proportion of time spent on session showing non-social stimuli: Significant difference (ASD > TD) c. Breakpoint of non-social video session (number of times the child clicked the non-social video before giving up): Significant difference (ASD > TD).
Carpenter et al. (2021) Specific measure: social-emotional reciprocity/facial expressions Country: USA	1. ASD group *M*_age_ (*SD*) = 26.2 (4.1) months, *N* = 22, %male = 77.27 2. TD group *M*_age_ (*SD*) = 21.7 (3.8) months, *N* = 74, %male = 58 3. DD (Non-ASD delay) *M*_age_ (*SD*) = 23.9 (3.7) months, *N* = 8, %male = 62	Tablet computer (iPad)	Task description: Same as Campbell et al. (2019). Set of developmentally appropriate videos presented on the screen. Social (woman singing nursery rhymes) and non-social (noise-making toy) videos were also presented on the left and right sides of the screen, respectively. Child response: Front camera captured child’s video. ML algorithm predicted the probability of facial expressions (positive, neutral or other) for each 3-s window. #trials: 1 Setting: Clinic Duration: 5 min	ASD group displayed increased frequency of neutral expression compared to the non-ASD group. 1. Area under the ROC curve (predict ASD diagnosis based on two metrics – mean probability of facial expressions for 3-s windows and frames not attending for each movie): (1) Bubbles_1: 0.75, Bunny: 0.81, Puppets: 0.78, Rhymes: 0.83 Bubbles_2: 0.79. Model for ‘Rhymes’ movie yielded the strongest predictive ability
Zhao and Lu (2020) Specific measure: Imitation of facial expressions Country: China	1. ASD group *M*_age_ (*SD*) = NA (NA) months; Range: 36–72 months, *N* = 10, %male = 50 2. TD group *M*_age_ (*SD*) = NA (NA) months; Range: 60–96 months, *N* = 10, %male = 50	Varying: mobile phones (both Android and iOS), personal computer (Windows 10) with Windows server	Task description: A software prompted children to imitate seven different facial expressions through pictures and sounds (happy, sad, angry, disgust, surprise, fear, neutral). Child response: Camera recorded children’s facial expressions. ML algorithm estimated probabilities of the child making each of the seven expressions. #trials: 3 per expression Setting: Quiet school room Duration: Not specified	1. Accuracy to correctly imitate on-screen expressions: Significant difference (ASD < TD). ASD group performance most compromised for disgust, neutral, surprise and fear.
Bovery et al. (2021) Specific measure: Social preference and attention Country: USA	1. ASD group *M*_age_ (*SD*) = 26.19 (4.07) months, *N* = 22, %male = 77.27 2. TD group *M*_age_ (*SD*) = 21.91 (3.78) months, *N* = 82, %male = 58.54 Includes eight children with a diagnosis of language delay or developmental delay sufficient to qualify for speech or developmental therapy	Tablet computer (iPad fourth generation). Front camera recorded video at 1280 × 720 resolution and 30 frames/s	Task description: 1-min video containing social (singing women) and non-social (dynamic toys with sound) stimuli. Both types of stimuli changed at pre-specified times within the movie (a different woman or toy appeared at different times). Child response: Webcam captured children’s videos while they watched the movie. ML algorithm 1 – automatically detected head position. ML algorithm 2 – predicted where on the screen the child looked (left, right or indeterminate) #trials: 1 Setting: Clinic Duration: 1 min	1. Attention on screen (number of frames in which child looked at the screen): Significant difference (ASD < TD). 2. Attention to social versus non-social: No difference 3. Attention shift to novel stimuli when stimuli changes: Significant difference (ASD < TD).
Lu et al. (2019) Specific measure: Abstract rule learning in social and non-social contexts Country: China	1. ASD group^a^ *M*_age_ (*SD*) = 69.96 (9.72) months, *N* = 28, %male = 82.14 2. TD group *M*_age_ (*SD*) = 66.36 (5.04) months, *N* = 28, %male = 82.14	Laptop computer (specifications not provided)	Task description: Gamified tasks based on distrust and deception tasks. Token hidden in one of three boxes. Player incorrectly indicated to opponent the box with the hidden token. Two conditions: non-social – computer as opponent, and social – computer-controlled avatar as opponent that children believed were real people. Games included (1) recognising and avoiding misleading cues (distrust) and (2) providing misleading cues (deception). Child response: Distrust task: correct response was to choose any of the two boxes not indicated by the computer (computer always indicated the wrong box). Deception task: correct response was to indicate a box other than the one in which token was hidden (Computer always chose the indicated box). #trials: 10 each Setting: Not specified Duration: Not specified	1. Number of correct trials: Distrust task – Significant difference (ASD < TD) in both social and non-social conditions. Deception task – Significant difference (ASD < TD) in social, but not in non-social condition. 2. Speed of learning: Significant difference (ASD < TD) in both tasks in social condition. No difference in non-social condition.
Nakai et al. (2014) Specific measure: Speech intonation Country: Japan	1. ASD group^a^ *M*_age_ (*SD*) = 87.23 (6.76) months, *N* = 26, %male = 76.92 2. TD group *M*_age_ (*SD*) = 83.48 (9.13) months, *N* = 37, %male = 54.05	Lavalier Microphone (Sony, ECM-77B/9X), Audio Capture (ROLAND, EDIROL UA-25EX), Mobile Note PC (TOSHIBA, Dynabook SS RX2/T7G)	Task description: 50 picture cards of animals and objects displayed on the screen. Child had to name them. Child response: Microphone fixed to child’s clothing recorded verbal responses. Audio data of correct responses isolated for analysis. #trials: 2 Setting: Not specified Duration: Not specified	1. Pitch coefficient of variation (each word): No difference in preschool children (4–6 years). Significant difference in school-age children (7–9 years). 2. Pitch range and SD (each word): No difference 3. Whole speech pitch metrics: No difference.
Wijesinghe et al. (2019) Specific measure: Speech characteristic Country: Sri Lanka	1. ASD group Age range in both groups: 18–36 months *M*_age_ (*SD*) = NA (NA) months, *N* = 8, %male = NA 2. TD group *M*_age_ (*SD*) = NA (NA), *N* = 9, %male = NA	Voice recorder (specifications not provided)	Task description: Voice recorder placed either within a pocket in the child’s clothing or within a metre from the child for periods varying from 2 to 10 h. Recording of conversations between the index child with a familiar adult in a familiar environment was captured. Child response: Audio signals were segmented as ‘silent’ and ‘non-silent’, and further to ‘vocal’ and ‘noise’ segments from non-silent segments. Child utterance data measured from vocal segments only. #trials: Not specified Setting: Child’s familiar environment Duration: 2–10 h	1. ML accuracy: ML model with feature set (duration of each utterance category (child uttering a meaningful word, meaningless word, vegetative sounds, adult utterances, noises, silences, total child duration and total audio duration per 10 min) not effective in classifying children).
Gyori et al. (2008) Specific measure: Social-emotional reciprocity/facial expressions Country: Hungary	1. ASD group *M*_age_ (*SD*) = 58.38 (8.45) months, *N* = 13, %male = 69.2 2. TD group *M*_age_ (*SD*) = 57.15 (6.74) months, *N* = 13, %male = 46.2	Desktop computer. Webcam below the monitor captured emotional expressions. Noldus FaceReader (v5.1, Noldus Information Technology) for emotional states analysis	Task description: Gamified task to assess ability to use deception and sabotage as social strategies in competitive and co-operative contexts. Game included tasks to evoke emotional, behavioural and gaze responses. Child response: Webcam captured child’s video; analysed by the Noldus FaceReader #trials: Not specified Setting: Laboratory Duration: ~24 min	1. Mean intensities of emotions: No difference
Lin et al. (2013) Specific measure: Language Country: Taiwan	1. ASD group^a^ *M*_age_ (*SD*) = 66.11 (8.90) months, *N* = 35, %male = NA 2. TD group *M*_age_ (*SD*) = 60 (12) months, *N* = 300, %male = 49	Computer. Online (worldwide web) language assessment tool connected to backend server. ‘Offline’ version available which allows temporary storage of data on device for later upload.	Task description: Six language tests presented in auditory and visual formats – (1) decoding (DE), (2) homographs (HOM), (3) visual vocabulary comprehension (VVC), (4) auditory vocabulary comprehension (AVC), (5) visual sentence comprehension (VSC) and (6) auditory sentence comprehension (ASC). Child response: Test administrator recorded correct/incorrect responses using keyboard key presses or mouse clicks. #trials: DE = 50, HOM = 14, VVC = 53, AVC = 38, VSC = 15, ASC = 15. However, 104/186 items retained in the final test. Setting: Quiet room (clinic or school) Duration: ~35 min	1. Accuracy on each sub-test: Significant difference in DE, HOM, VVC, VSC (ASD > TD) and ASC (ASD < TD). No difference in AVC. In DE and VVC, largest difference seen at 4 years (ASD > TD), reduces at 6 years. For ASC, differences at 4 years (ASD < TD) further enhanced at 6 years.
Chaminade et al. (2015) Specific measure: Biological motion/anthropomorphic bias Country: France	1. ASD group *M*_age_ (*SD*) = 61 (25) months, *N* = 12, %male = 91.6% 2. TD group *M*_age_ (*SD*) = 39 (7) months. In total, 12 of the youngest TD children mentally age-matched to ASD sample, *N* = 24, %male = 58.3%	Computer touchscreen (specifications not provided)	Task description: Gamified task where four characters (two humans and two cartoons) are associated with two kinds of motions: biological and artificial. From these set of combinations, two videos were presented simultaneously on the left and right of the touchscreen. Child response: Children touched the video they liked more. The touched video grew in size while the other disappeared. #trials: 16/session, up to 4 sessions/participant. Setting: Laboratory Duration: Not specified	1. Proportion of choices to biological motion (human): Significant difference (ASD < TD) 2. Proportion of choices to biological motion (cartoon): No difference. Results similar for mentally and chronologically age-matched samples
Deschamps et al. (2014) Specific measure: Empathy and prosocial behaviour Country: The Netherlands	1. ASD group^a^ *M*_age_ (*SD*) = 81.59 (6.95) months, *N* = 22, %male = 81.82 2. TD group *M*_age_ (*SD*) = 86.39 (6.71) months, *N* = 29, %male = 82.76	Computer screen (specifications not provided)	Task description: Ball-throwing computer game against two computer-controlled players who gave rewards when ball was passed to them. When rewards ran out (final round), one player showed progressively distressed facial expressions each time the ball was not passed to them. Two conditions – girl and boy as the distressed player. Child response: Choose player to pass ball. #trials: 20 in final round, 10 in previous rounds Setting: Quiet school room Duration: Not specified	1. Number of ball throws to distressed player: No difference
Aresti-Bartolome et al. (2015) Specific measure: Social interaction and eye contact Country: Spain	1. ASD group^a^ *M*_age_ (*SD*) = NA. Age range: 36–96 months, *N* = 20, %male = NA 2. TD group *M*_age_ (*SD*) = NA. Age range: 36–96 months, *N* = 20, %male = NA Gender-matched to the ASD group	21″ touch screen. Tactile pointer of 40 cm. Games configured by APNABI Association	Task description: Gamified task with three levels of difficulty involving collecting as many pre-specified items within a 3-min period as possible by touching the screen with a pointer. Game stopped every 30 s or when an error was made. Child response: For game to continue, child had to interact with test administrator, who recorded the latency of the interaction through key presses on the keyboard (separate keys for interactions with and without eye contact). #trials: 1 per level Setting: Classroom Duration: 12 min	1. Latency of interaction with administrator: Significant difference (ASD > TD). 2. Number of interactions with eye contact: Significant difference (ASD < TD) 3. Task completion (% not completing levels): 10, 14, 15% in ASD versus 0, 0, 5% in TD 4. Number of pre-specified items collected per level: Significant difference (ASD < TD)
P. Li et al. (2016) Specific measure: Selective trust Country: China	1. ASD group^a^ *M*_age_ (*SD*) = 73.55 (9.71) months, *N* = 30, %male = 86.67 2. TD group *M*_age_ (*SD*) = 70.31 (7.79) months, *N* = 30, %male = 86.67	Computer screen (specifications not provided)	Task description: Virtual candy hidden in one of the two boxes. Boxes had pictures of faces with contrasting themes: own versus other race, attractive versus unattractive, trustworthy versus not trustworthy Child response: Tap on box with preferred face characteristic. #trials: 30 trials, 10 per condition. Setting: Not specified Duration: Not specified	1. Accuracy of choosing own race, more attractive, or more trustworthy faces: (a) Race: No difference; (b) Attractiveness: Significant difference (ASD > TD) for more attractive face; (c) Trustworthiness: No difference
Borsos and Gyori (2017) Specific measure: Social-emotional reciprocity/facial expressions Country: Hungary	1. ASD group *M*_age_ (*SD*) = 58.38 (8.45) months, *N* = 13, %male = 69.2 2. TD group *M*_age_ (*SD*) = 57.15 (6.74) months, *N* = 13, %male = 46.2	Desktop computer. Webcam below the monitor captured emotional expressions. Noldus FaceReader (v5.1, Noldus Information Technology) for emotional states analysis Standard desktop-mounted eye-tracking device (EyeFollower 2 by LC Technologies) to detect attention to screen.	Task description and child response: Same as Gyori et al. (2008). For each frame, data were classified as invalid if the Noldus FaceReader was unable to identify the face or unable to assign an emotion to the frame. #trials: 3 game sections played one time each Setting: Laboratory Duration: ~24 min	1. Mean of static (i.e. frame-by-frame) intensities of emotional states: Significant difference in scared and surprised emotions (ASD > TD). 2. Speed of emotion expression change: Significant difference in ‘surprised’ emotion (ASD > TD). 3. Valid data ratio (ratio of valid vs invalid data): No significant difference.
Martin et al. (2018) Specific measure: Social preference and head postural response Country: USA	1. ASD group *M*_age_ (*SD*) = 60.8 (16.52) months, *N* = 21, %male = 80.95 2. TD group *M*_age_ (*SD*) = 51.23 (15.35) months, *N* = 21, %male = 66.67	19″ video monitor with a camera mounted on the top edge	Task description: Six videos containing social and non-social stimuli presented on a screen. One video each was purely non-social and purely social. Remaining four had a mix of both. Child response: Camera recorded children’s videos while they observed the stimuli. A computer vision algorithm (Zface) tracked pitch, yaw and roll of head movement frame-by-frame. #trials: 1 Setting: Laboratory Duration: 16 min	1. Proportion of successfully tracked frames: No significant difference 2. Number and duration of epochs (successfully tracked consecutive frames) per video: No significant difference 3. Angular displacement: Significant difference in yaw (ASD > TD) in social video. No difference for pitch and roll. No difference between groups in non-social video. 4. Angular velocity: Significant difference in yaw and roll in social video (ASD > TD), but no difference in pitch. No difference between groups in non-social video.
Li et al. (2020) Specific measure: False-belief understanding Country: China	1. ASD group^a^ *M*_age_ (*SD*) = 77.04 (9) months, *N* = 17, %male = 82.4 2. TD group *M*_age_ (*SD*) = 77.04 (9) months, *N* = 17, %male = 58.82	Notebook computer (specifications not provided)	Task description (1): Truth value judgement (TVJ) task: One puppet made statements about pictures shown on a screen. Statements contained factive (‘knows’), counter-factive (‘pretend’) and non-factive (‘thinks’) verbs. Child response: Judge the truth of the puppet’s statement by pressing one of the three buttons – ‘yes’, ‘no’ and ‘maybe’. # trials: 10 ‘pretend’ and 15 (‘knows’, ‘thinks’) Task description (2): First- and second-order false-belief tasks presented as series of pictures with voiceovers. Child response: Answer questions to demonstrate FB understanding. Each correct answer awarded one point. #trials: 8 Setting: Kindergarten/primary school/training centre Duration: Not specified	1. Mean (*SD*) of TVJ task score: Significant difference (ASD < TD) for ‘pretend’ condition; approaching significance (ASD < TD) in ‘know’ condition. No difference in ‘think’ condition. 2a. Mean (*SD*) of first-order FB tasks: Significantly different (ASD < TD) 2b. Mean (*SD*) of second-order FB tasks: Significantly different (ASD < TD)
Li et al. (2019) Specific measure: Social gaze Country: China	1. ASD group *M*_age_ (*SD*) = NA (NA) months; Range: 48–84 months, *N* = 136, %male = NA 2. TD group *M*_age_ (*SD*) = NA (NA) months; Range: 72–96 months, *N* = 136, %male = NA	Computer screen (specifications not provided). Head movements recorded using 360 intelligent camera	Task description: Children seated in front of a screen with their mother’s picture displayed. Child response: Camera recorded children’s videos while they observed the stimuli. ML approach used to track trajectory of eye movements in the first 2001 frames of the video. Trajectory data divided into angle and length information and used to classify children as ASD or TD. #trials: 1 Setting: Primary and special education schools Duration: 10 min	1. No. of frames with eyes not visible: Significant difference (ASD > TD) 2. Accuracy of ML algorithm to classify children into diagnostic groups: 92.6% using all features (both length and angle information)
Shahab et al. (2017) Specific measure: Joint attention and imitation Country: Iran	1. ASD group^a^ *M*_age_ (*SD*) = 58.8 (9.96) months, *N* = 14, %male = NA 2. TD group *M*_age_ (*SD*) = 60 (10.8) months, *N* = 21, %male = NA	Virtual-reality (VR) setup	Task description: VR setup to play the xylophone and drums, led by robots controlled remotely via an operator. Step 1: Virtual robot showed child how to play a virtual drum and the xylophone, using VR controllers as mallets. Step 2: Children asked to describe what they saw in the virtual room. In case of unable to name objects present in the room, robots pointed to objects to direct the child’s attention. Midway through task, children requested to wear VR headset. Child response: Imitate robot’s actions. Child behaviour recorded by two video cameras. One point awarded for naming each picture in virtual room (4 total). #trials: 1 Setting: Laboratory Duration: ~ 10 min	1. Score in the drum and xylophone imitation tasks: Significant difference (ASD < TD). 2. Picture naming: Significant difference (ASD < TD) 3. Task engagement (defined as (1) duration engaged with the game and (2) duration for which the child wore the VR headset): Significant difference (ASD < TD) for both metrics.
Jyoti et al. (2020) Specific measure: Joint attention Country: India	1. ASD group^a^ *M*_age_ (*SD*) = 76.8 (14.76) months, *N* = 20, %male = 65 2. TD group *M*_age_ (*SD*) = 80.4 (10.38) months, *N* = 20, %male = 65	VR-enabled HCI-based task platform. Touch-sensitive monitor to record child responses.	Task description: Joint attention (JA) task with 3D avatar within a VR setup. The avatar provided cues through (1) eye gaze alone; (2) head turn with gaze; (3) finger pointing, head turn and gaze and (4) all of the above and sparkling of the target contingent with JA cues. JA cues offered randomly. Child response: Touching the target object indicated by JA cue on a touch-sensitive monitor. Performance recorded on confirmation of choice. #trials: 8 (2 per cue) Setting: Laboratory Duration: 20 min	1. Average performance score on JA task: Significant difference (ASD < TD) in two cues – eye gaze alone and eye gaze with head turn. Ceiling effect (100%) for TD group irrespective of JA cue type. ASD group performance improved with increasing information in the JA cue. 2. Average reaction time: Significant difference (ASD > TD). Approximately three times longer in ASD. Average response time decreases as cue information increases. 3. Effectiveness index (EI): EI = PI (scaled performance score) + (1-RTI) (scaled reaction time). Significant difference (TD > ASD). EI consistently > 1.5 across all cues for TD. For ASD group, EI progressively increases from 0.5 to 1.5 for JA cues with increasing information.
DSM-5 criteria: Stereotypical, repetitive or restricted behaviours or interests				
Moradi et al. (2017) Specific measure: Patterns of play movements Country: Iran	2.ASD group^a^ *M*_age_ (*SD*) = 57.24 (12.36) months, *N* = 25, %male = 84% 2. TD group *M*_age_ (*SD*) = 60.24 (8.76) months, *N* = 25, %male = 64%	Wii remote (with a 3-axis Micro-Electro-Mechanical System (MEMS) ADXL330 accelerometer) embedded into a toy car	Task description: Children played with a toy car embedded with an accelerometer. Child response: Accelerometer recorded car’s movement in 3D. Data transferred using Bluetooth or Wi-Fi. Typical data file consisted of ~5000 samples of time and acceleration data in 3D representing child’s play with the car. #trials: 1 Setting: quiet room Duration: ~5 min	1. Accuracy, sensitivity, specificity of ML (SVM) methods: The full feature set (44 – see select examples below) discriminated between groups with reasonable accuracy (62%), sensitivity (65%) and specificity (61%). Feature examples: Play time; In each three dimension – correlation of acceleration between two of the three axes, mean and variance of acceleration, dominant frequencies of acceleration direction, total acceleration signal energy, number of jolts in the forward direction.
Motor (not covered in DSM-5 criteria)				
Rafique et al. (2019) Specific measure: Fine-motor drawing movements Country: Pakistan	1. ASD group *M*_age_ (*SD*) = NA (NA) months, Age range in both groups: 60–144 months, *M*: 89 months, *N* = 22, %male = 77.2 2. TD group *M*_age_ (*SD*) = NA (NA) months, *N* = 22, %male = 54.5	Android phone (version 6.0.1)	Task description: Trace and colour a dotted square shape. Smartphone was placed on a flat table, so that the sensor values recorded force applied on the phone. Smartphone recorded four inertial and six touch data. Child response: Perform drawing task. #trials: Not specified Setting: School room Duration: Not specified	1. Accuracy of ML methods to classify children into diagnostic groups using top 10 features of the creativity game reported by Anzulewicz et al. (2016): Accuracy > 85%.
Mahmoudi-Nejad et al. (2017) Specific measure: Fine-motor tracing movements Country: Iran	1. ASD group *M*_age_ (*SD*) = NA (NA) months. Range: 48–84 months, *N* = 5, %male = 100% 2. TD group *M*_age_ (*SD*) = NA (NA) months. Age-matched with ASD group, *N* = 7, %male = 57.14%	Tablet computer and smartphone (specifications not provided)	Task description: Follow a pre-specified path marked with pink flowers to take a bee to its hive. Pink flower turns green (win) when bee touches it. Untouched flowers turned red (fail). Haptic feedback provided if defined trajectory is not followed. Child response: Trace path to take bee to hive. Touch data recorded on the tablet. Children awarded 0–4 stars/trial depending on their accuracy (win/total). Data analysed only from Level 1 since ASD group could not play beyond this level. #trials: 8 sub-levels within 4 difficulty levels. Setting: Not specified Duration: Not specified	45 features extracted from touch data in two categories – point-based and progress-based. Points-based: Calculated using two adjacent points the child touched (e.g. distance, velocity, acceleration, time, curvature, error) Progress-based: Indicator of attempts made (e.g. score, levels played, completion time). ML methods (Linear SVM) used to classify children into their respective groups. Three features – Total score, Average velocity and Average curvature – discriminated between ASD and TD groups.
Dawson et al. (2018) Specific measure: Rate of head movements Country: USA	1. ASD group *M*_age_ (*SD*) = 26.19 (4.07) months, *N* = 22, %male = 77.27 2. TD group *M*_age_ (*SD*) = 21.91 (3.78) months, *N* = 82, %male = 58.54 Includes eight children with a diagnosis of language delay or developmental delay sufficient to qualify for speech or developmental therapy.	Tablet computer (iPad). Front camera recorded video at 1280 × 720 resolution and 30 frames/s	Task description: Same as Campbell et al. (2019). Child response: Camera captured child video while they viewed stimuli on tablet screen. ML algorithm detected and tracked head position. Three head pose angles – yaw (left-right), pitch (up-down) and roll (left-right tilt) calculated per frame. Child was considered to be looking at the screen when yaw ⩽ 20° #trials: 1 Setting: Clinic Duration: 5 min	1. Rate ratio of head movement of ASD with TD as reference: Rate ratio calculated from the association between head movement rate (for every one-third second across the movie) and ASD diagnosis. Robust differences observed in four out of five movies (except bubbles) (ASD > TD). These four movies had complex stimuli compared to bubbles video. Associations unchanged with eight DD participants removed from the TD group during sensitivity analysis.
Fleury et al. (2013) Specific measure: Fine-motor drawing movements Country: Canada	1. ASD group^a^ *M*_age_ (*SD*) = 81.6 (19.08) months, *N* = 15, %male = 86.96 2. TD group *M*_age_ (*SD*) = 63 (12) months, *N* = 19, %male = 85	Wacom Cintiq 15X digitising tablet and pen	Task description: Draw circles on a touchscreen using a stylus. Six conditions based on hand-use (dominant vs non-dominant) and three circle drawing styles (continuous drawing without pausing: comfortable vs fast pace; and discontinuous by pausing at the top before starting again). Child response: Draw circles as per the six specified conditions. #trials: 1 each (6 total) Setting: Not specified Duration: 14 min	1. Mean circle drawing time: No difference 2. Coefficient of variation (CV) of circle drawing time: Significant difference in discontinuous condition (ASD > TD) 3. Power spectral density (PSD), root mean squared (RMS) fluctuations during drawing and statistical persistence (Hurst coefficient): No difference
Dowd et al. (2012) Specific measure: Motor planning and execution Country: Australia	1. ASD group^a^ *M*_age_ (*SD*) = 74.39 (16.8) months, *N* = 11, %male = 72.73 2. TD group *M*_age_ (*SD*) = 79.19 (18) months, *N* = 12, %male = 75	17″ LCD touch screen (MicroTouch 3MM170), connected to a HP Compaq 6910p laptop	Task description: Point-to-point movement task. Join two points presented on a vertical plane using a stylus. Start position at the bottom centre of the screen. End position at the top of the screen either left, centre or right from start. Some trials had a visual distractor near the target. Child response: Trace a line from start to end. Stylus movement was sampled at 125 Hz. #trials: 25 Setting: Laboratory Duration: Not specified	1. Various kinematic variables: a. Significant difference in variability of movement preparation time (ASD > TD). No difference in any other variable b. Interaction effects observed for distractor condition: longer and more variable total movement time in TD. No difference in ASD.
Crippa et al. (2013) Specific measure: Hand-eye coordination Country: Italy	1. ASD group^a^ *M*_age_ (*SD*) = 74.4 (25.2) months, *N* = 14, %male = 85.7% 2. TD group *M*_age_ (*SD*) = 75.6 (27.6) months, *N* = 14, Age- and gender-matched	100 Hz 17″ LCD touch screen (Elo TouchSystem) at 1024 × 768 pixel resolution. 50X Tobii for eye-tracking, AB Danderyd, Sweden, with 5-point calibration. Video recorder	Task description: Gap overlap task. Child response: Two conditions – (1) ‘press’: participants indicated the target’s left/right position on screen using a button box; and (2) ‘touch’: participants touched the targets on the screen. Movements started from a set position by lifting the hand off a pad on the table. #trials: 3 sessions of 16 trials each. Setting: Laboratory Duration: Not specified	1. Reaction time for button press in ‘press’ condition: No difference 2. Accuracy of touching target in ‘touch’ condition: No difference 3. Eye-hand coordination (Pearson correlation between eye fixation latency on target and hand response (key presses and touch times): Strong and significant correlation in TD. Weak (‘press’) or no (‘touch’) correlation in ASD.
				4. Differences in gap and overlap conditions: No difference in ‘press’ condition. Significant gap effect in TD but not ASD in ‘touch’ condition.
Jung et al. (2006) Specific measure: Visuomotor coordination Country: South Korea	1. ASD group^a^ *M*_age_ (*SD*) = 72 (0) months, *N* = 12, %male = 83.33 2. TD group Age range: 60–72 months, *N* = 20, %male = NA, *M*_age_ (*SD*) = NA	VR setup: Pentium IV PC, one projector, one screen (200 X150 cm), one infrared reflector, one digital camera and tangible devices (stick)	Task description: Burst virtual balloons in a VR setup. Auditory and visual reinforcements provided when successful. Number of balloons and the type of reinforcement changed by level. Child response: Burst virtual balloons by moving a real stick. #trials: 10 sessions Setting: Laboratory Duration: Not specified	1. Accuracy: No difference (may be due to high variability in ASD group). 2. Reaction time: Significant difference (ASD > TD) 3. Movement of stick to pop balloons: Significant difference (ASD < TD) 4. PCA index: Significant difference indicating TD more efficient in popping balloons using combination of three variables.
Alcañiz Raya et al. (2020) Specific measure: Gross-motor movements Country: Spain	1. ASD group^a^ *M*_age_ (*SD*) = 61.56 (16.2) months, *N* = 24, %male = 87.5 2. TD group *M*_age_ (*SD*) = 58.32 (10.92) months, *N* = 25, %male = 64	RGB-D camera (includes depth information in video recordings): RealSense – camera D435 (FRAMOS, Munich, Germany) and Intel RealSense SDK 2.0 (Intel RealSense Technology, Santa Clara, CA, USA) VR setup: CAVE-Automatic Virtual Environment (CAVETM): semi-immersive room with 3–6 rear-projected surfaces	Task description: VR setup showing street intersection with three types of stimuli conditions: visual (V): avatar walks into the scene and waves to participant; visual–auditory (VA): avatar dances to a song for 10 s; visual–auditory–olfactory (VAO): two avatars bite into a buttered muffin, while participant is able to smell, see and hear actions. Child response: Imitate avatars while a camera recorded movements. OpenPose algorithm used to detect body joints in each frame. Joint displacement computed across two consecutive frames and then averaged across a condition. #trials: 9 (3 per condition) Setting: Laboratory Duration: 14 min	1. Average movement of each joint in each condition: Significant difference (ASD > TD) in all three conditions. Condition V: leg, head and trunk Condition VA: leg and head Condition VAO: head ML models used to discriminate between TD and ASD groups. 2. ASD classification accuracy: Head metrics performed the best in all conditions. Highest accuracies observed when using head metrics in VAO condition and all joints in V condition (89.36%). Least accuracy (70.2%) in model including all joints and all conditions.
Cognitive (not covered in DSM-5 criteria)				
Chen et al. (2019) Specific measure: Executive functioning Country: China	1. ASD group^a^ *M*_age_ (*SD*) = 54 (11) months, *N* = 40, %male = NA 2. TD group *M*_age_ (*SD*) = 55 (7) months, *N* = 51, %male = NA. Groups were gender-matched.	Tablet computer (PC or PAD)	Task description: Series of gamified tasks presented on a screen. Included joint attention tasks, responding to social requests, matching shapes, categorisation, visual search and visuomotor coordination. Child responses: Tap on target objects on the screen during gameplay. Responses stored on the tablet. One point awarded for each correct answer. #trials: 1 Setting: Therapeutic centre and kindergarten Duration: 15–20 min	1. Completion rate (proportion of participants completing game): Significant difference (ASD < TD for children > 48 months) 2. Efficiency (ratio of average score to average completion time): Significant difference (ASD < TD; visual search and visuomotor coordination in ⩽ 48 months only; Categorisation and matching shapes in > 48 months)
Hetzroni et al. (2019) Specific measure: Relational (abstract) thinking Country: Israel	1. ASD group^a^ *M*_age_ (*SD*) = 78.84 (10.44) months, *N* = 24, %male = 75 2. TD group *M*_age_ (*SD*) = 67.56 (3.24) months, *N* = 24, %male = 41.66 3. IDD group *M*_age_ (*SD*) = 142.56 (27.48) months, *N* = 24, %male = 62.5	Portable computer (specifications not provided)	Task description: Pictures of animals presented in various relational configurations (example – two koalas as mirror images). In a subset of trials, another panel with the same configuration, but with a different animal, was shown to strengthen concept. Influence of level of familiarity determined using local (known), foreign (partially known) and made-up (unknown) animal pictures. Child response: Tap on one of the two options that match the configuration of the target panel. Correct responses awarded one point. #trials: 8 Setting: quiet room Duration: 30–40 min	1. Accuracy (proportion of correct choices): Significant difference (IDD < ASD < TD in single panel condition). No benefit of second panel (to strengthen concept) to ASD group. 2. Influence of familiarity: Performance unaffected by familiarity in ASD group. In the TD and IDD groups, better performance with known target images.
Veenstra et al. (2012) Specific measure: Executive functioning Country: The Netherlands	1. ASD group^a^ *M*_age_ (*SD*) = 61.2 (9.6) months, *N* = 13, %male = NA 2. TD group *M*_age_ (*SD*) = 45.6 (1.68) months, *N* = 5, %male = NA	Computer screen (specifications not provided)	Task description: Web-based gamified Go/No-go task (www.samenslim.nl). Child response: Through mouse clicks during gameplay. #trials: 2–3 sessions, max of 7 games/session. Setting: quiet room in a medical day-care centre or playgroup Duration: Not specified	Significant differences observed in all metrics: 1. Accuracy: ASD < TD 2: No-go (Number of clicks when no clicks should have been made): ASD < TD 3. Missing go (No clicks during clicking moments): ASD > TD 4. Go (Number of clicks during clicking moments): ASD < TD 5. Reaction time: ASD > TD 6. Repeated clicks (Repeated clicks on the same objects): ASD > TD 7. Variability across levels: ASD < TD
Gardiner et al. (2017) Specific measure: Executive functioning Country: Canada	1. ASD group *M*_age_ (*SD*) = 66.88 (13.41) months, *N* = 24, %male = 83.33 2. TD group *M*_age_ (*SD*) = 58.47 (15.87) months, *N* = 19, %male = 57.89	Touchscreen monitor (specification not provided)	Task description: Computerised tasks assessing executive functioning: 1. spatial working memory (Boxes task) 2. cognitive flexibility, inhibition and working memory (Go/No-Go, Preschool-Continuous Performance Test (PCPT)) 3. Multicomponent planning task (Monkey Tower) – adaptation of Tower of Hanoi task Child response: Tap on screen during gameplay #trials: 1 Setting: Laboratory Duration: 90–120 min	1. Accuracy on Boxes, Go/No-Go, PCPT: No difference 2. Number of correct trials in multicomponent planning task: Approaching significance (ASD < TD; *p* = 0.036).

ASD: autism spectrum disorder; TD: typically developing; AUC: area under the ROC curve; ROI: region of interest; RT: reaction time; DD: developmental delay; ROC: Receiver Operating Characteristic; FB: false belief understanding; PI: performance index (scaled); RTI: reaction time index (scaled); PCA: Principal Component Analysis; IDD: Intellectual and Developmental Disabilities.

The following colour coding has been used in the column named ‘Device specifications’ to indicate the feasibility of the device for use in low-resource settings: Green = most feasible; Red = least feasible.

**Colour coding**
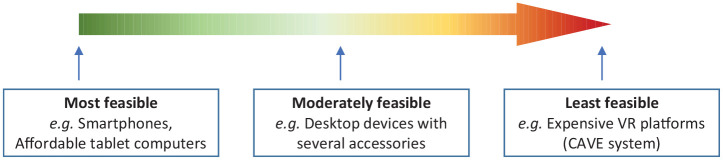

Figure added to Table 1 legend: Colour coding to indicate feasibility of administering the identified digital tools in low-resource settings.

The time taken to complete the assessment, when specified in the article, is included in the column titled ‘Experimental setup’ in red font colour, along with the number of trials of the assessment and the experimental setting (laboratory, clinic, school, home, etc).

a To maintain consistency, age when reported in years was converted to months by multiplying by 12.

### Risk of bias

A list of questions was compiled from two risk of bias assessment tools – Joanna Briggs Institute Critical Appraisal tools: Checklist for Case–Control Studies (Joanna Briggs Institute, 2017) and the QualSyst tool: Checklist for assessing the quality of quantitative studies from the Alberta Heritage Foundation for Medical Research Health Technology Assessment Initiative Series (Kmet et al., 2004). Some questions from the compiled list were adapted; the final set of questions used is listed in Supplementary Table 3.

### Community involvement statement

As the reported study is a review *a posteriori* of the reported research, there was no community involvement.

## Results

### Study selection

**Figure 1.**
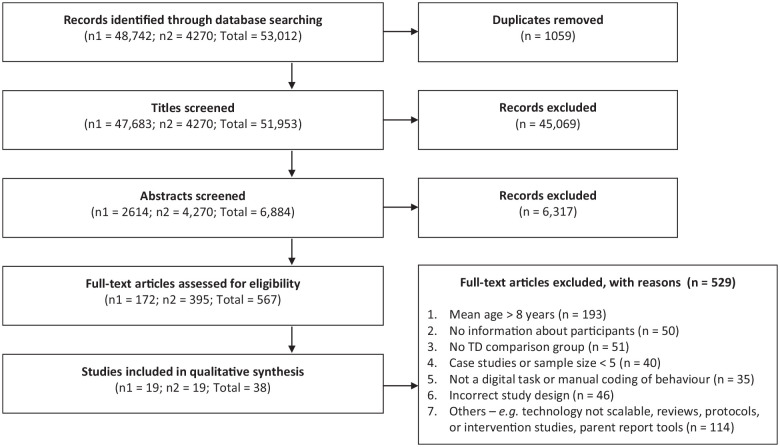
Study selection flow diagram. Articles were identified and screened into two phases (number of articles in Phases 1 and 2 are represented as n1 and n2, respectively). During Phase 1 conducted in May 2018, no date filters were applied. The search strategy was broad and aimed to capture articles describing digital tools used for risk identification of children with developmental delays and two neurodevelopmental disorders (ASD and ADHD). Given the large number of articles identified in Phase 1, a decision was taken to focus this review only on scalable digital tools (see definition in the text) for ASD risk detection. When this search was updated in Phase 2 in October 2020, the search strategy was modified to reflect the restricted focus. A date filter was applied to select only those articles published between June 2018 and October 2020 during Phase 2. Title and abstract screening was done simultaneously when updating the search.

A total of 51,953 titles and 6884 abstracts were screened for relevance across the two phases (Figure 1). However, 567 full-text articles were screened for eligibility, of which 38 met inclusion criteria. The most common reason for exclusion was the age criterion (mean age > 8 years; *n* = 193). Other reasons for exclusions were the following: the primary focus of the article was feasibility testing, protocol description or delivery of an intervention (*n* = 114); absence of a TD comparison group (*n* = 51); sample size of less than five in at least one group (*n* = 40); task not administered on digital devices or child responses being coded manually (*n* = 35) (Figure 1).

### Description of the study participants

**Figure 2.**
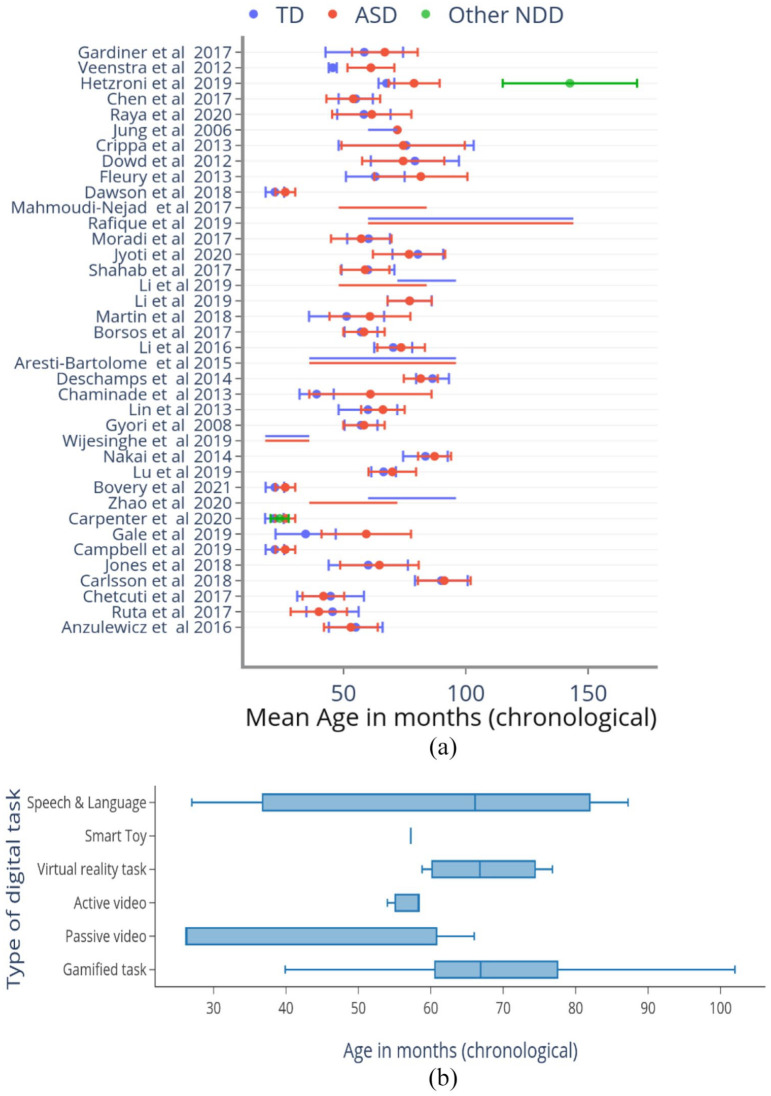
Age distribution of participants across studies and types of tasks applied to them. (a) Mean (dot) and standard deviation (error bar) of the chronological age (CA) of participants in months is represented on the X-axis (red = ASD; blue = TD; green = neurodevelopmental disorders not including ASD). Y-axis lists the included studies (in the order presented in Table 1). Most studies used CA-matched samples, except for Hetzroni et al. (2019) in which the other NDD group was significantly older as a result of being developmentally age-matched to the ASD and TD groups. Some studies reported the range of one or more participant groups instead of the mean and SD. In those cases, the range was represented as a horizontal line (e.g. Zhao et al., 2020; 10th row) using the same colour scheme. (b) Box plot demonstrating the age group to which different types of tasks were applied. The vertical line within the box represents the median age of participants to which the tasks were administered. The whiskers represent the 25th and 75th percentiles of participant age, respectively.

Together, these studies analysed results from 889 ASD participants, 1348 TD participants and 32 participants with a neurodevelopmental disorder other than ASD (intellectual disability (*N* = 24), developmental delay or language delay (*N* = 8)). The proportion of males in the ASD group (79.3%) exceeded that in the TD (60.4%) or NDD (62.3%) comparison groups. The mean age of the ASD group was also higher (65.3 months) compared to the TD group (60.9 months). Among 31 studies reporting the mean age, the TD comparison group was younger than the ASD group in the majority of studies (21/31; 67.7%), to allow the groups to be developmentally age-matched (Table 1 – Participant details and Figure 2). In all but three papers (Bovery et al., 2021; Gale et al., 2019; Ruta et al., 2017), the participants were > 36 months of age, indicative of most tools’ applicability beyond infancy and toddlerhood (Figure 2(a)). Gamified tasks, tasks presented on virtual-reality (VR) platforms and those assessing speech and language, were applied to children with mean age > 5 years. Tasks involving video recording of children’s behaviours while they interacted with stimuli presented on a screen or with the experimenter were typically applied to children with mean age below 5 years (Figure 2(b)). It is to be noted that developmental age equivalents and individual skill profiles and preferences are more significant than chronological ages in determining applicability.

### Overview of scalable digital tools for early assessment of ASD risk

This is an emerging field, 28 of the 38 included articles (73.6%) having been published in the past 5 years (Supplementary Figure 2). The tools identified were predominantly at the pilot or ‘proof-of-concept’ stage and typically administered in laboratory or clinic settings by research staff. Studies demonstrated high levels of heterogeneity across tasks used to assess diagnostic discriminative ability, the type of technology used to implement them, primary metrics evaluated and developmental domains assessed. Tasks were presented on portable technologies, such as laptops (H. Li & Leung, 2020; Lu et al., 2019), tablet computers (Anzulewicz et al., 2016; Bovery et al., 2021; Campbell et al., 2019; Carlsson et al., 2018; Carpenter et al., 2021; Chen et al., 2019; Chetcuti et al., 2019; Dawson et al., 2018; Fleury et al., 2013; Gale et al., 2019; Jones et al., 2018; Mahmoudi-Nejad et al., 2017; Ruta et al., 2017), smartphones (Mahmoudi-Nejad et al., 2017; Rafique et al., 2019; Zhao & Lu, 2020), intelligent toys (Moradi et al., 2017) and digital audio recorders (Nakai et al., 2014; Wijesinghe et al., 2019), and non-portable technologies, such as desktop computers (Aresti-Bartolome et al., 2015; Borsos & Gyori, 2017; Chaminade et al., 2015; Crippa et al., 2013; Deschamps et al., 2014; Dowd et al., 2012; Gardiner et al., 2017; Gyori et al., 2018; Hetzroni et al., 2019; J. Li et al., 2020; P. Li et al., 2016; Lin et al., 2013; Martin et al., 2018; Veenstra et al., 2012) and VR platforms of varying sophistication (Jung et al., 2006; Jyoti & Lahiri, 2020; Alcañiz Raya et al., 2020; Shahab et al., 2017).

In total, 21 studies (55.3%) used gamified tasks (Anzulewicz et al., 2016; Aresti-Bartolome et al., 2015; Carlsson et al., 2018; Chaminade et al., 2015; Chen et al., 2019; Chetcuti et al., 2019; Crippa et al., 2013; Deschamps et al., 2014; Dowd et al., 2012; Fleury et al., 2013; Gale et al., 2019; Gardiner et al., 2017; Hetzroni et al., 2019; Jones et al., 2018; P. Li et al., 2016; H. Li & Leung, 2020; Lu et al., 2019; Mahmoudi-Nejad et al., 2017; Rafique et al., 2019; Ruta et al., 2017; Veenstra et al., 2012), making these the most common type of performance-based tasks to detect autism risk in early childhood. Other types of assessments included video recording of children’s behaviours while they viewed or interacted with stimuli presented on a screen (*n* = 9; 23.7%) (Borsos & Gyori, 2017; Bovery et al., 2021; Campbell et al., 2019; Carpenter et al., 2021; Dawson et al., 2018; Gyori et al., 2018; J. Li et al., 2020; Martin et al., 2018; Zhao & Lu, 2020), tasks using VR platforms (*n* = 4; 10.5%) (Jung et al., 2006; Jyoti & Lahiri, 2020; Alcañiz Raya et al., 2020; Shahab et al., 2017) and audio recording of children’s speech (*n* = 2; 5.2%) (Nakai et al., 2014; Wijesinghe et al., 2019). One study used a toy car with an embedded accelerometer to record the child’s movement characteristics while they played with the toy (Moradi et al., 2017).

**Figure 3.**
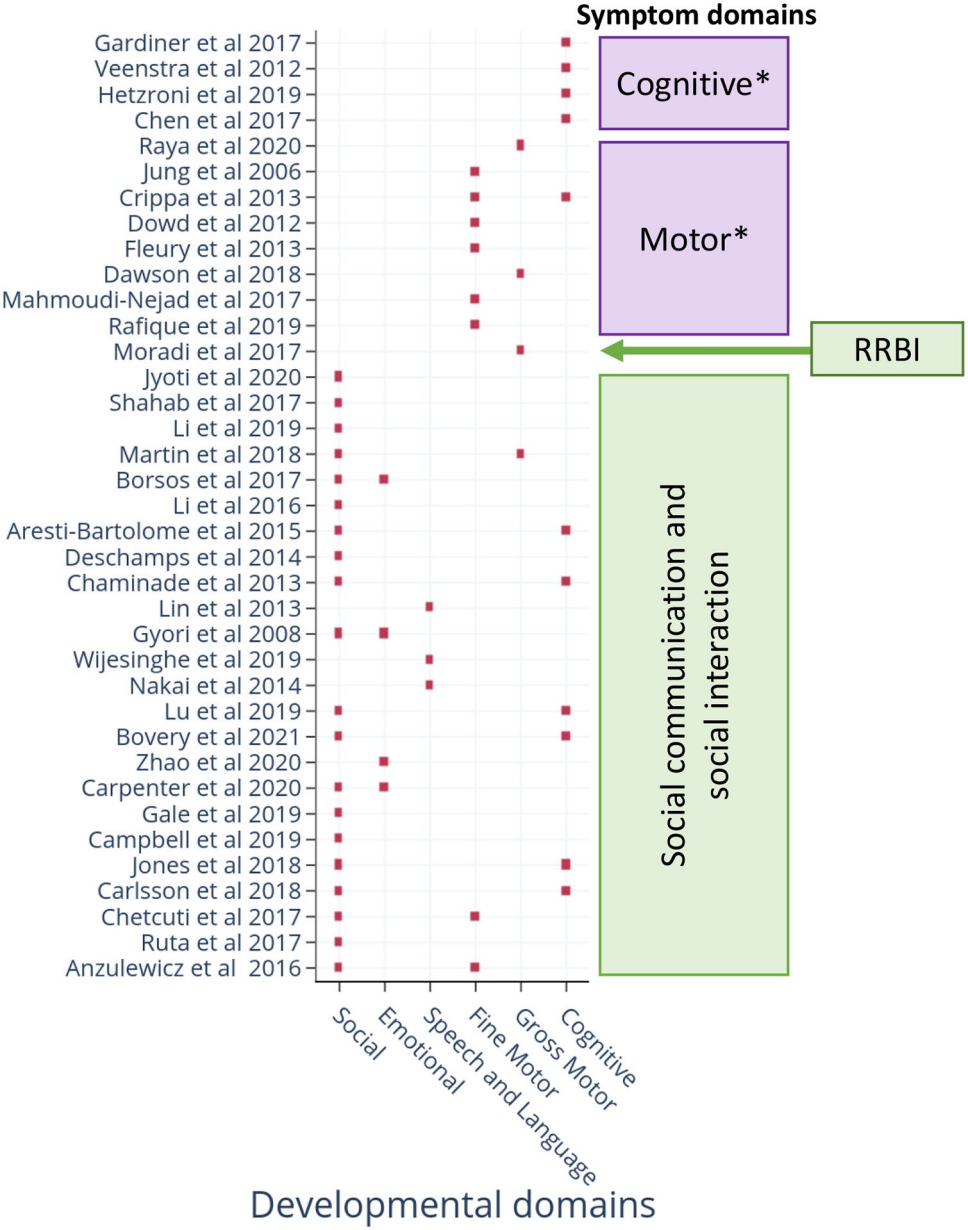
DSM-5 criteria and developmental area(s) assessed by scalable digital tools. The X-axis represents the developmental domains targeted for ASD risk assessment. Y-axis lists the included studies (in the order presented in Table 1). The criteria covered by the studies are indicated on the right-hand side. Green boxes (social communication/social interaction; RRBI: repetitive or restrictive behaviours or interests) are part of the DSM-5 criteria. Purple boxes (motor and cognitive) are **not** part of the DSM-5 criteria for ASD diagnosis, hence marked with an ‘*’ symbol.

Together, these technologies targeted both criteria set within the DSM-5 for ASDs (Table 1). Several technologies also assessed neurodevelopmental domains not included within the DSM-5 criteria, but known to be affected in many children with ASD. Examples include deficits in motor and cognitive abilities (Figure 3). Two papers included a non-ASD NDD comparison group in the study design (Carpenter et al., 2021; Hetzroni et al., 2019); both demonstrated specificity to ASD symptoms. Eight studies (21.1%) used machine learning (ML) to identify nonlinear combinations of metrics as discriminants (Bovery et al., 2021; Campbell et al., 2019; Carpenter et al., 2021; Dawson et al., 2018; J. Li et al., 2020; Martin et al., 2018; Alcañiz Raya et al., 2020; Zhao & Lu, 2020).

The majority of studies were conducted in high-income countries (26/38); however, three recent studies from India (Jyoti & Lahiri, 2020), Pakistan (Rafique et al., 2019) and Sri Lanka (Wijesinghe et al., 2019) and three from Iran (Shahab et al., 2017; Moradi et al., 2017; and Mahmoudi-Nejad et al., 2017), all low- and middle-income countries (LMICs), represent an encouraging trend for global mental health research.

### Assessment of the risk of bias

**Figure 4.**
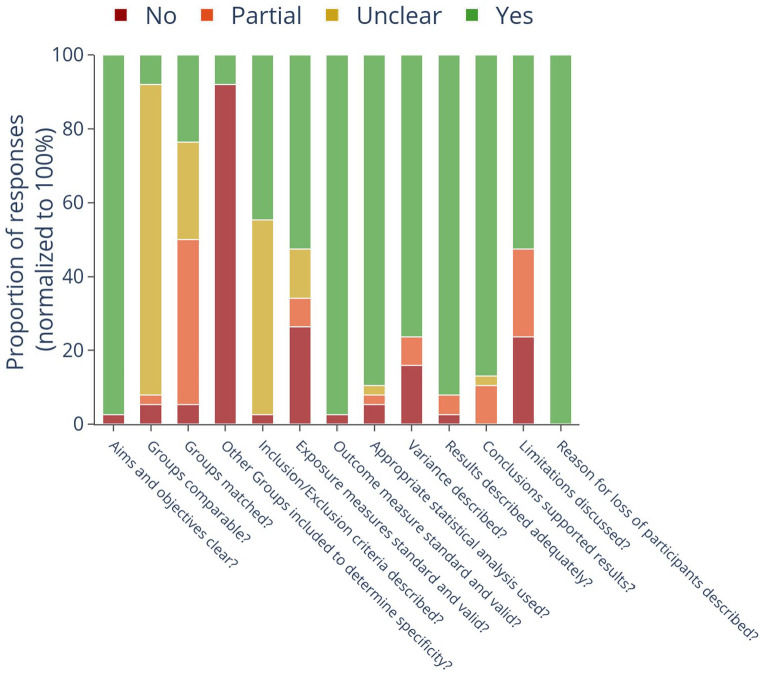
Risk of bias of included studies. The X-axis lists the parameters used to assess the risk of bias of the included studies. Y-axis represents the proportion of studies classified into the four groups, normalised to 100%. In addition to Yes (green)/No (red) responses to each question, responses were also classified as partial (orange: some, but not all criteria met) and unclear (brown: no data available to make a decision).

More than 90% of the included articles (34 of 38) clearly described the aims and objectives, details of implementation and used valid statistical methods to report their results (Figure 4). They also reported the reasons for the loss of participants when applicable, and the results supported the conclusions. However, participant demographic details were only reported by 3/38 (7.9%) papers (Supplementary Table 2: Additional participant details), which omission precluded the determination of adequate matching of participant characteristics across groups in a case–control study design. However, 12/38 (31.6%) papers did not describe the study setting or population from which the TD group was recruited. Groups were adequately matched on gender and developmental age only in 9/38 (23.7%) studies. Gender distribution across groups was largely mismatched, with the ASD group typically having a greater proportion of males compared to the TD group (Table 1). In total, 21/38 studies did not clearly describe the inclusion/exclusion criteria for participant recruitment across both the groups. Whereas, 17/38 articles used standardised diagnostic or screening tools to select participants in the ASD group. Meanwhile, 36/38 papers did not include an NDD group without ASD to demonstrate the specificity of the tasks and metrics to ASD symptoms. Finally, while all the included studies were at the proof-of-concept stage, limitations related to small sample sizes, lack of generalisability and inadequate matching of samples were described only in 20/38 (52.6%) studies (Figure 4). Of note, none of the included articles explicitly reported on any measure related to reliability (intra- and inter-assessor reliability, test–retest reliability) or validity (face, construct, content and criterion). While greater understanding of each tool’s validity and reliability is important for their ultimate use, this state of development is to be expected for an emerging field, as the main focus of these initial studies is to demonstrate feasibility and explore the discriminative ability of these tools.

### Characteristics of digital ASD assessment tools

Detailed characteristics of individual tasks, details of implementation and their discriminative ability as reported by the studies are presented in Table 1. Detailed description and comparisons of tasks used to address the primary research questions are presented in the Supplementary material. Based on the evidence, the potential of these tools to screen for autism risk in low-resource settings is discussed below.

#### Tasks using portable technologies (tablet computers, smartphones, toy cars and digital audio recorders)

All tasks using mobile technology could be completed in 8 min on average (range = 1–20 min). Except for tasks assessing accuracy on executive functioning skills (Chen et al., 2019; Jones et al., 2018), all other tasks and metrics could discriminate between ASD and TD at a group level (details in Supplementary material). Tasks tapping the social and motor domains were particularly reliable, as discriminative ability was demonstrated by a total of 13 studies led by different study groups using a variety of tasks, metrics and devices. This included seven tasks tapping the social domain (Bovery et al., 2021; Campbell et al., 2019; Carlsson et al., 2018; Gale et al., 2019; H. Li & Leung, 2020; Lu et al., 2019; Ruta et al., 2017) encompassing social versus non-social stimulus preference and theory-of-mind, and six tapping fine- and gross-motor domains (Anzulewicz et al., 2016; Chetcuti et al., 2019; Dawson et al., 2018; Fleury et al., 2013; Mahmoudi-Nejad et al., 2017; Rafique et al., 2019). Also within the social domain, two studies assessed group differences in facial expressions in two ways – evoked expressions while watching animated videos (Carpenter et al., 2021) versus imitating the facial expressions presented on the screen (Zhao & Lu, 2020). Therefore, while similar data capture and analysis methods were used and significant group differences were reported by both, a direct comparison of the tasks and metrics for this particular construct was not possible. One study each used a toy car and digital audio recorder to assess autism risk. The former was moderately successful while the latter failed – however, more replications of these tasks are required to determine their utility.

#### Tasks using non-portable technology (desktop computers and VR platforms)

Tasks presented on desktop computers were highly heterogeneous in terms of the ASD phenotype assessed, making it difficult to synthesise results across studies. Except for three studies assessing EF, all others assessed a unique skill using different tasks and metrics. Consistent with tasks presented on mobile devices, accuracy on EF tasks showed mixed results in the desktop technology format as well. Results related to reaction time were consistent, with the ASD group reported to be slower in providing responses in EF tasks. Most tasks tapping the social (Aresti-Bartolome et al., 2015; Chaminade et al., 2015; Martin et al., 2018) and motor (Crippa et al., 2013; Dowd et al., 2012) domains continued to demonstrate significant group differences. The two studies assessing speech and language (Lin et al., 2013; Nakai et al., 2014) used very different metrics to assess group differences (pitch characteristics vs accuracy), so no comparison was possible. Similarly, the results from facial expression analysis using the Noldus FaceReader (Borsos & Gyori, 2017; Gyori et al., 2018) were too preliminary to determine their utility for use as autism screening measures.

The VR format of ASD risk assessment, although showing promising results, depended on sophisticated devices and administration in laboratory settings by trained research staff. However, the discriminative ability of these tasks tapping joint attention, motor imitation and visuomotor coordination continues to highlight the promise of the social and motor domains to identify autism risk. Tasks using desktop technology were completed in 23 min on average, about thrice as long as those on portable devices (8 min). VR tasks took 14.6 min on average (see Supplementary material).

## Discussion

### Tasks can be brief, portable and largely automated

This study identifies and characterises digital tools that have the potential to be applied in direct assessment of autism risk in early childhood in low-resource settings. Because the availability of skilled human resources is a major limitation in these settings (Divan et al., 2021), we focused on tools that require minimum assessor judgement during administration, and whose data analysis could be automated with no to minimal manual inputs. Two main modalities of direct child assessments were identified – gamified tasks and video or audio recordings of the participant while they viewed/responded to stimuli on the screen or VR platforms, or interacted with research staff or family members. Tasks were presented on both portable and non-portable technologies, namely laptops, tablet computers and smartphones on the one hand, and desktop computers and VR platforms on the other. However, some tasks presented on non-portable technologies but requiring child responses on touchscreens, or in which children’s videos were captured using webcams, are easily adaptable to portable devices (Jyoti & Lahiri, 2022). The majority of the assessments were administered in laboratory or clinic settings, but some were also deployed in homes, schools and daycares. While trained research staff administered these tasks in all studies, they typically provided only simple instructions and demonstrations before participants became able to engage with the tasks independently – extending these tasks’ promise in the hands of non-specialists. Finally, tasks delivered on portable technologies could be completed in less than 10 min on average, and most others within 30 min. Therefore, once validated, the types of tasks identified and their potential to be delivered on low-cost devices by non-specialists pave a promising path for ASD risk detection in low-resource settings, which bear the largest burden of cases worldwide (Baxter et al., 2015). Six recent studies’ being based in LMICs is an encouraging trend towards this direction.

### Social and motor skills discriminate best

While specific tasks and metrics targeted multiple developmental domains, those tapping social and motor domains were the most promising. Most studies assessing these domains reported significant group differences using a variety of technologies, tasks and metrics. Lower preference for social stimuli emerged as one of the most reliable metrics. The ASD group consistently preferred non-social stimuli irrespective of the format in which these were presented, be it static images or videos presented on tablet or desktop screens. This general result of aversion to social stimuli aligns with the literature, including decades of eye-tracking literature demonstrating reduced time spent looking at social stimuli in the ASD group (Papagiannopoulou et al., 2014). Individual studies reported unique tasks and metrics relevant to the social domain that successfully discriminated between the ASD and TD groups; examples include anthropomorphic bias and the applied theory-of-mind ability to deceive and to distrust opponents. The ASD group was also found to take longer to orient to the person calling their name, or to initiate social interactions. These are examples of digital tasks tapping literature-backed autism-relevant phenotypes that historically have been assessed by in-person interactions with trained staff (Baron-Cohen et al., 1985; Bruinsma et al., 2004; Federici et al., 2020; Zwaigenbaum et al., 2005). Their success in digital and gamified formats is encouraging for their potential to scale as screening tools in low-resource settings.

Similarly, a variety of tasks tapping the motor domain found consistent differences between the ASD and TD groups. Tasks assessing fine-motor abilities, which were largely administered on portable technology including tablets and smartphones, found differences in the pattern of interactions with smart devices. Kinematic analyses contrasting autistic versus non-autistic touchscreen movements in gamified tasks have demonstrated greater variance in speed and direction with longer, less straight movement paths, amid a less fluid, more piecewise movement style (Weisblatt et al., 2019) and greater spatiotemporal error across a range of tasks (Dubey et al., 2021). A challenge in quantifying visuomotor behaviour is the choice of specific derived metrics from a large number of possible ones, for example, acceleration, jerk, direction, variances and maxima thereof, duration and extent of movement. An important element of the future research agenda will be to combine motor and developmental literatures so as to give the autism field a standard set of parameters with which movements can be described and compared across studies. Discriminative results reviewed here focus thematically on inter-trial variability in response duration in motor planning tasks, accuracy in imitation of complex motor gestures, and visuomotor coordination, but the devil is in the details of specific derived measures.

However, studies assessing gross motor movements were typically based on video recordings of child behaviour while viewing on-screen stimuli or imitating avatars presented on VR platforms. The most consistent result was differences in head movements, indicative of poor postural head control in the ASD group. These results are consistent with the literature on autism-related motor phenotypes, for example, stride length variability (Kindregan et al., 2015), and differences across a range of behaviours including arm movements, gait (Lum et al., 2021), postural stability and oculomotor coordination (Fournier et al., 2010; Johnson et al., 2016), all of which are suggestive of an overall deficit in motor planning (Rinehart et al., 2006) and coordination. Mechanisms proposed for these observed autistic deficits are an inability to chain together sequential motor events (Cattaneo et al., 2007; von Hofsten & Rosander, 2012) and difficulty incorporating visual error feedback in an online movement process (Haswell et al., 2009).

Another consistent discriminating metric was slower reaction times in the ASD group, measured as the latency to respond selectively to pre-specified target objects. This slowing manifested in a range of tasks including tablet-based gamified tasks assessing executive functioning, VR-based tasks of joint attention and visuomotor coordination, and video recording of child behaviour to assess latency in orienting to name. The validity of this metric is supported by the literature demonstrating slower reaction times in older children (Herrero et al., 2015) and adults (Lartseva et al., 2014; Schmitz et al., 2007; van den Boomen et al., 2019) with ASD using computerised simple and choice reaction tasks. Although pathologically slowed reaction time can flag developmental issues in general, by itself it would be too blunt an instrument to discriminate autism from other neurodevelopmental conditions. Similarly, task completion and task engagement, the latter defined variably as number of frames in which an ML algorithm was able to compute relevant metrics from children’s facial landmarks, or the duration for which the child played the game, were found to be a useful discriminating metric in six different studies, but cannot point to autism in particular.

### Accounting for heterogeneities, sex differences, ages and stages, and available resources

Arriving at a set of screening tasks that can discriminate autism in all its forms and presentations is not as simple as deciding which tasks work. One needs to know, rather, which tasks work for which subtypes of this heterogeneous condition, at what ages and developmental stages, and in which real-world circumstances dependent on culture and context. Given the early stage of development, most studies are limited in their generalisability to broader samples and contexts. The absence of demographic details in the majority of papers not only precludes determining the comparability between the ASD and TD groups within and across studies but also obscures our understanding of the heterogeneity of the study samples and the subpopulations for whom these tools are applicable. For instance, motor tasks might be especially predictive in some individuals who are not beginning to speak on time (Belmonte et al., 2013), and conversely, assays of social responsiveness might be more predictive in others. Furthermore, while gender specificities in ASD prevalence and symptoms exist (Halladay et al., 2015), gender differences were not addressed in any of the studies. Therefore, further confirmatory studies are required to test the validity, reliability and specificity of these tools before they can be deployed as screening measures. One group has already started planning Phase 3 trials using large samples in different contexts (Millar et al., 2019), which is an important stride in the right direction. The US Food and Drug Administration (FDA) recently authorised marketing of the Cognoa ASD Diagnosis Aid, software using ML algorithms to help predict the risk of autism based on parent reports, videos of child behaviour and health provider inputs, as an adjunct though not a substitute to the regular diagnostic process. A similar open-source effort has shown > 78% accuracy in discriminating between autism, intellectual disability and typical development in 2- to 7-year-old toddlers in low-resource settings (Dubey et al., 2021).

These studies demonstrate great potential pending further validation. All social and motor tasks met our minimum criteria for use in low-resource settings – (1) independent of assessor judgement and hence with the potential to be easily administered by non-specialist providers and (2) capturing data in digital format that could be objectively analysed. Furthermore, they could be completed in less than 20 min, largely on easily accessible and affordable devices. The potential for portability of some of the tasks administered on non-portable technology (desktops, VR) is high since most tasks were in a format that could be adapted to portable technologies (e.g. Jyoti & Lahiri, 2022). With respect to the type of tasks, computer vision analysis of child behaviour is more versatile as it is applicable to the full spectrum of children of varying ages and abilities. Gamified tasks, however, the majority identified by our search, are only suitable for older or less impaired children who can understand and follow game instructions.

### Advantages of digital tools for ASD risk assessment and implications for global health research

These novel tools are uncovering nuanced differences in child behaviour using objective and automated measures. Examples include inter-trial variability of a few seconds during motor planning, and differences in force, pressure and patterns of tap-and-drag gestures in tablet-based gamified tasks. The Research Domain Criteria framework theorises that early deviations from normative developmental trends may be predictive of later disorders (Cuthbert, 2014, 2020), including autism (Tunç et al., 2019). Digital tools provide the most feasible method to develop global normative trends of ASD-relevant phenotypes, which could be used to flag children with differential trajectories. Their potential to be administered by non-specialists make them amenable to task-sharing and stepped-care approaches, paving the way for large-scale ASD screening across diverse locations including low-resource settings. Given the current state of development of the tools, magnitude of demand and heterogeneity within the ASD population, digital tools can potentially aid in the initial screening of autism risk at scale. However, a confirmatory diagnosis should only be made by a clinician.

Notwithstanding their potential, it is important to recognise that a ‘digital divide’ currently exists between high- and low-resourced settings, the developmental benefits of technological advances disproportionately accruing to educated and resource-rich communities that have access to smart devices, adequate power supplies and the know-how of Internet services (World Bank, 2016). Therefore, the feasibility of using various types of digital platforms for ASD risk assessment must be carefully considered with respect to the specific LMIC context and setting in which they will be used. Portable computers and mobile devices provide the highest levels of accessibility, affordability and potential for scale across all settings of the global South. Therefore, tools that are adapted for delivery on these platforms would be most feasible to use across all settings and ensure that the technological advances do not inadvertently increase the digital divide in the global ASD community (Kumm et al., 2022).

### Methodological considerations to advance the field

Based on the key findings and limitations discussed above, we provide the following directions for future studies. Tasks tapping the social and motor domains that are available in gamified formats or that estimate metrics using computer vision analysis, and provide objective measures analyzable using standard and machine learning methods, should be prioritised for further development. The main goal should be to further validate these tasks and metrics using prospective cross-sectional study designs and larger samples, and to standardise derived measures across tasks. Studies should focus on establishing reliability (intra- and inter-assessor reliability, test–retest reliability) and validity (face, construct, content and criterion) of these tools and report these metrics in future publications. Larger samples would allow assessing heterogeneity using dimensional measures (Stevens et al., 2019) and refining ML algorithms when applicable. Once a tool or task is validated for a single population, its acceptability, feasibility and validity should be assessed in diverse settings and geographies, checking the consistency of psychometric properties across contexts. Once metrics describing reliability, validity, sensitivity and specificity are available for these tools, we can begin to make judgements about their use as screening or diagnostic tools.

For this reason of cultural context, especially, stakeholder families and community members must be involved in the design and execution of such research (Staniszewska et al., 2018), ideally with representation on the research team itself. Stakeholder and community involvement heightens recruitment and retention in general (Crocker et al., 2018), and in autism studies in particular (McKinney et al., 2021), makes tools globally relevant, especially to low-resource and underserved communities (Witham et al., 2020) and must be an integral part of the research design process from the stage of conceptualisation, rather than left as an afterthought. None of the studies reviewed has reported a strategy for stakeholder and community involvement. This must change.

The social and motor tasks currently being administered on desktop computers and VR platforms should be translated for delivery on portable devices to improve access, and then subjected to feasibility and validation testing in different settings riding the wave of widespread mHealth technology use worldwide (Abaza & Marschollek, 2017; Osei & Mashamba-Thompson, 2021). Since research is linked to local capacity building (Durkin et al., 2015), greater testing of these tools in diverse low-resource settings will also generate the necessary awareness and skills, in the community and in researchers, to build momentum towards universal screening of children’s development. Finally, longitudinal studies should be designed to evaluate the developmental trajectories using metrics of the most promising tools and to develop a deeper understanding of the normative trends of ASD-relevant phenotypes.

We identified a few standalone studies that assessed ASD-related behaviours using very specific and unique tasks that could significantly discriminate between groups. Examples include studies assessing evoked and imitated facial expressions, and speech and language. We recommend replication of these studies by different groups in different populations to further test their validity and reliability.

**Box 1.** Considerations for future studies evaluating digital tools for ASD risk assessments.

**1. Specificity to developmental delay in general, not to autism in particular:** Although the addition of other NDD comparison groups can demonstrate digital assessment tools’ specificity to ASD symptoms, at the stage of community screening and referral, it may be impractical to focus specifically on autism risk alone: a child who is at risk of a developmental delay but not autistic still needs a referral. **2. Participant characteristics:** Future studies should report participants’ demographic characteristics in detail. Studies would also benefit from using standardised measures to rule out ASD symptoms in the TD group, especially as we move towards more dimensional and nuanced measures proposed to characterise heterogeneity within groups. Mental age-matched TD comparison groups should be included in studies involving ASD participants with comorbid ID. **3. Choice of device:** While Android devices could be prioritised since they are cheaper and more widely available in LMICs, care must be taken to ensure that the sensitivity and accuracy of the device-derived metric is appropriate for the task delivered on the device, and that the delivery of stimuli and collection of responses will be robust to the fast pace of hardware development and marketing. **4. Individual risk measures:** Finally, while group differences are adequate to evaluate the potential of these novel technologies, the analytical methods should be refined to allow quantifying individual risk. Bayesian classification and ML methods have been employed by a few studies reviewed. Discriminative features from multiple developmental domains could serve as individual features in an ML algorithm designed to predict autism risk (Dawson & Sapiro, 2019; Jin et al., 2015; Kang et al., 2020; Liu et al., 2016). This multidimensional strategy would be akin to the standard practice of observing multiple behaviours for ASD diagnosis. Therefore, while any one of the features may not be enough to capture the full heterogeneity of the spectrum, the combination as determined through an ML approach may achieve higher degrees of sensitivity or specificity. **5. Ecological validity:** Taking advantage of rapid advancements in computer vision, future assessments should focus on computing social and motor metrics relevant to the autism phenotype from brief, automated tasks portable into homes, schools or other ecologically valid settings. Some examples include reciprocal social interactions, repetitive behaviours and sensory sensitivity during regular interactions of the child with their peers, teachers and parents in home or school settings or during solitary play, captured using cameras on tablet computers or smartphones. **6. Patient and public involvement:** Stakeholders and community members (e.g. community health workers) must be involved in planning and execution of the research, from the beginning.

ASD: autism spectrum disorder; TD: typically developing; NDD: neurodevelopmental disorders; ID: Intellectual Disability.

### Limitations

As this review was limited to case–control studies, digital tools piloted or validated using other types of study designs will have been missed. Second, since we only included peer-reviewed published articles in English, emerging technologies that may have been presented in conferences or in other languages are not included. Third, the search was last updated in October 2020. This review does not cover new tools and new data (including some of our own) published beyond this date, posing a limitation in view of the rapid pace at which new technologies are introduced and evaluated in this dynamic area of research.

## Conclusion

This review identifies and characterises digital tools for direct observational assessment of autism risk in early childhood that have the potential to scale in low-resource settings. This characterisation encompasses tasks and their associated metrics, developmental domains assessed, discriminative ability and details of implementation. Tasks assessing social and motor domains were found to be particularly promising and reliable in discriminating between ASD and TD groups. Their implementation on readily accessible technologies – half of them on portable devices, such as tablet computers and smartphones – coupled with objective output measures make them suitable for task-sharing with non-specialist providers. Novel methods, such as computer vision and ML, are increasingly being coupled with these tasks to allow for objective and automated analysis of data, leading to more in-depth and nuanced understanding of ASD symptoms and furthering their potential for task-sharing approaches and identification of autism risk at the individual level. The time is ripe for the field to move beyond pilot studies and small samples to large-scale, multinational validation studies, using prospective cross-sectional or longitudinal designs conceived, developed and implemented in collaboration with stakeholders and communities.

## Supplemental Material

sj-pdf-1-aut-10.1177_13623613221133176 – Supplemental material for Digital tools for direct assessment of autism risk during early childhood: A systematic review Click here for additional data file. Supplemental material, sj-pdf-1-aut-10.1177_13623613221133176 for Digital tools for direct assessment of autism risk during early childhood: A systematic review by Debarati Mukherjee, Supriya Bhavnani, Georgia Lockwood Estrin, Vaisnavi Rao, Jayashree Dasgupta, Hiba Irfan, Bhismadev Chakrabarti, Vikram Patel and Matthew K Belmonte in Autism
