# Supplementary material for: Digital tools for direct assessment of autism risk during early childhood: A systematic review
Source: Autism. 2022 Nov 7;28(1):6–31. doi: 10.1177/13623613221133176 (PMC10771029; doi:10.1177/13623613221133176)

Supplement to Mukherjee et al. "Digital tools for direct assessment of autism risk during early childhood: A systematic review"

These data and discussion are integral to the manuscript and are placed in this supplement only to accommodate the limit on length of the main text.

Characteristics of digital ASD assessment tools

Detailed characteristics of individual tasks, details of implementation and their discriminative ability as reported by the studies are presented in Table 1. In this section, we present detailed results for the following primary research questions, categorized by the type of technology – i) Description of tasks and neurodevelopmental domains assessed; ii) Discriminative ability of the primary task metrics; and iii) Experimental set-up and details of implementation.

1) Portable technology

1.1) Tablet-computers and smartphones

17 studies (44.7%) used laptops, tablet computers or smartphones. While the majority using tablet computers used Apple iPads (53.3%) (Anzulewicz et al., 2016; Bovery et al., 2018; Campbell et al., 2018; Carpenter et al., 2021; Chetcuti et al., 2019; Dawson et al., 2018; Jones et al., 2018; Ruta et al., 2017), a few studies also used Android devices (Gale et al., 2019; Rafique et al., 2019).

Gamified tasks

12 (70.6%) studies used gamified tasks to measure social preference (Gale et al., 2019; Ruta et al., 2017), false belief understanding (Carlsson et al., 2018; H. Li & Leung, 2020), deception and deceit (Lu et al., 2019), executive functioning (Chen et al., 2019; Jones et al., 2018), and fine motor abilities (Anzulewicz et al., 2016; Chetcuti et al., 2019; Fleury et al., 2013; Mahmoudi-Nejad et al., 2017; Rafique et al., 2019). Gamified tasks presented on tablet-computers and smartphones required simple demonstrations of the task by the test administrator before the child could independently engage with them. Children provided responses directly on the screen through tap and drag gestures, or by using a stylus, and data were automatically recorded and stored in the device. Two studies additionally used data from the tablet's accelerometer and gyroscopes to record forces being input into the device (Anzulewicz et al., 2016; Rafique et al., 2019).

Social preference: Two studies assessed social preference using gamified tasks, both administered on tablet computers (Gale et al., 2019; Ruta et al., 2017). Social and non-social stimuli were presented directly side-by-side on the screen, or contingent on button presses. Social stimuli were images or videos of people or animals. Non-social stimuli were images of toys or abstract visual patterns. The primary metric was proportion of taps on the non-social stimuli or corresponding buttons, as a proxy for children's preference for those stimuli. The ASD group made a higher proportion of choices for the non-social stimuli compared to the TD group (Gale et al., 2019; Ruta et al., 2017). In reinforcement tasks where children had to tap on specific stimuli several times (increasing across trials) to access them, the ASD group tapped on the non-social stimuli significantly more times to view them compared to the TD group (Gale et al., 2019).

False belief understanding: One study (Carlsson et al., 2018) used a tablet-based gamified version of the Sally-Anne task (Baron-Cohen et al., 1985), while another presented a series of pictures on a laptop screen along with voiceovers (H. Li & Leung, 2020) to assess false-belief understanding. The primary metric in both the studies was accuracy in imputing another person's thoughts. This focus on accuracy distinguishes gamified false-belief evaluation in autism from that in the general population where accuracy is near ceiling and response latency (Paul et al., 2021) or mouse-tracking metrics can be more informative. The ASD group was found to

be less accurate than the TD group in false-belief understanding. Additionally, only 75% of the ASD participants completed the game in the first study (Carlsson et al., 2018), compared to 100% in the TD group.

Distrust and deceit: One study (Lu et al., 2019) presented a gamified task on a laptop to assess the ability of ASD and TD groups to distrust (avoid misleading cues) and deceive (provide misleading cues) a computer opponent to gain rewards. The primary metrics were accuracy (proportion of trials in which the child successfully deceived or distrusted the opponent) and the number of trials required to learn the correct response in the game. The ASD group was less accurate in deceiving and distrusting the opponent, especially when they falsely perceived the opponent to be a real person, and took significantly more trials to learn the correct responses required to win the game.

Executive functioning: Two studies used tablet-based gamified tasks to assess executive functioning (EF), specifically matching shapes, categorization, visual search and response inhibition (Chen et al., 2019; Jones et al., 2018). Primary metrics were accuracy, reaction time (latency to first response) or efficiency (ratio of average score to average completion time). One study discriminated groups based on reaction time, the ASD group being significantly slower compared to the TD group (Jones et al., 2018). Conflicting results were reported for the accuracy metric, with one study showing no group differences (Jones et al., 2018), the other showing reduced accuracy in the ASD group (Chen et al., 2019). The mean age of children in the second study (Chen et al., 2019) was slightly lower (55 months) than in the former study (60-64.6 months).

Fine motor: Five studies used tablet- and smartphone-based tasks to assess two kinds of motor abilities - motor planning and control (Anzulewicz et al., 2016; Fleury et al., 2013; Mahmoudi-Nejad et al., 2017; Rafique et al., 2019) and motor imitation (Chetcuti et al., 2019). The ASD group was found to be compromised in both.

For example, pause times in a discontinuous circle drawing task were significantly more variable across trials in the ASD group compared to the TD group (Fleury et al., 2013). Two studies (Anzulewicz et al., 2016; Rafique et al., 2019) using a trace and colour task on different device types (tablet vs smartphone) found greater mean impact force and gesture pressure in the ASD group, as well as greater use of distal parts of the screen and shorter dragging durations. Accuracy in a motor following task (Mahmoudi-Nejad et al., 2017) and a task requiring motor imitation of complex gestures (Chetcuti et al., 2019) was lower in the ASD group.

Video recording of child behaviour

Four studies from one group (Bovery et al., 2018; Campbell et al., 2018; Carpenter et al., 2021; Dawson et al., 2018) used the front camera on the tablet computer to record videos of children's behaviors while they watched age-appropriate videos containing social and non-social stimuli. Machine learning (ML) algorithms were used to automatically detect head position using coordinates of several facial landmarks, benchmarked to the distance from the screen. These head position metrics were subsequently used to estimate a variety of metrics related to the social and motor domains:

Social preference and orienting to name: ML algorithms were used to estimate the time children viewed social vs non-social stimuli presented on the left- and right-hand sides of the screen (Bovery et al., 2018), and the consistency and latency of head turns towards an assessor calling the child's name from behind (Campbell et al., 2018). No overall group differences were observed in looking time to social vs non-social stimuli (Bovery et al., 2018), in contrast to results reported earlier (Gale et al., 2019; Ruta et al., 2017). Compared to the TD group, the ASD group was found to be less consistent and took longer to orient towards the person calling their name (Campbell et al., 2018). Both these studies assessed differences in overt task engagement as a discriminating metric, defined as the number of frames in which the eyes or faces of children seated in front of a screen could be tracked by an automated algorithm. The ASD group was significantly less overtly engaged in both the tasks compared to the TD group.

Gross motor: ML algorithms were also used to estimate the rate of head movements in children while they watched an age-appropriate video, as a measure of postural head control (Dawson et al., 2018). The ASD group was found to have higher rates of head movement, indicating lower levels of postural control of the head (Dawson et al., 2018).

Facial expressions: Machine learning methods using features from facial landmarks were also used to estimate the type of facial expression made (positive, neutral, other) in response to animated videos presented on the tablet's screen (Carpenter et al., 2021). While watching videos meant to elicit emotions, a higher frequency of neutral expressions was reported in the ASD group. Another study used machine learning to predict the accuracy of *imitating* facial expressions presented on tablet or smartphone screens (Zhao & Lu, 2020). The ASD group was less accurate in imitating facial expressions, especially those of disgust, surprise, fear, and neutral expressions.

1.2) Toys and digital audio recorders

Intelligent toy car: One study used a toy car implanted with an accelerometer to record its motion in 3 dimensions while the child played with it (Moradi et al., 2017). Data, which comprised accelerations along with their timestamps for the duration of play, could be transferred to a computer or an Android device using Bluetooth or wifi technology. The primary metric was the accuracy of a ML algorithm to predict children's diagnostic classification based on the recorded (acceleration in 3 dimensions with timestamps) and derived (for example: duration of play, correlations between acceleration in two dimensions) data. These data were expected to capture repetitive and/or stereotypical movements often observed in children with autism (American Psychiatric Association, 2013). The algorithm discriminated

between the ASD and TD children with moderate accuracy (62%), sensitivity (65%), and specificity (61%). This task took 5 minutes to complete, and was administered in a quiet room in the presence of a research staff who gave minimal instructions.

Digital audio recorder: One study used a portable digital audio recorder that was placed either in a pocket in the child's clothing or within a meter of the child to record conversations between the index child and other family members (Wijesinghe et al., 2019). The recorder was left with the family for varying durations of 2-10 hours. Data comprised child's utterances segmented out from the entire conversation, which were subsequently used as features in a ML algorithm along with derived variables (for example: total duration, number of segments containing meaningful and meaningless words) to classify children into their diagnostic groups. The algorithm was not effective in discriminating between groups.

While the majority of these tasks (9/17; 52.9%) were administered in laboratory or clinic settings (Bovery et al., 2018; Campbell et al., 2018; Carlsson et al., 2018; Carpenter et al., 2021; Chetcuti et al., 2019; Dawson et al., 2018; Fleury et al., 2013; Gale et al., 2019; Jones et al., 2018), seven (41.2%) were also administered in the home or school (Carlsson et al., 2018; Chen et al., 2019; Chetcuti et al., 2019; Gale et al., 2019; H. Li & Leung, 2020; Rafique et al., 2019; Zhao & Lu, 2020), and four (23.5 %) were conducted in multiple settings (Carlsson et al., 2018; Chen et al., 2019; Chetcuti et al., 2019; Gale et al., 2019).

2) Non-portable technology

2.1) Desktop computers

15 studies (39.5%) used desktop computers to present gamified tasks (Aresti-Bartolome N. et al., 2015; Chaminade et al., 2015; Crippa et al., 2013; Deschamps et al., 2014; Dowd et al., 2012; Gardiner et al., 2017; Hetzroni et al., 2019; P. Li et al., 2016; Lin et al., 2013; Nakai et al., 2014; Veenstra et al., 2012) or video-record

children's behaviors (J. Li et al., 2020; Martin et al., 2018) and expressions (Borsos & Gyori, 2017; Gyori et al., 2018) while they watched or interacted with stimuli presented on the screen.

Gamified tasks

Nine (60%) studies used a variety of gamified tasks to test a range of functions relevant to the ASD phenotype. These included executive functioning (Aresti-Bartolome et al., 2015; Gardiner et al., 2017; Veenstra et al., 2012), abstract thinking abilities (Hetzroni et al., 2019), 'own-vs-other' preference (Li et al., 2016), prosocial behavior (Deschamps et al., 2015), anthropomorphic bias (Chaminade et al., 2015), motor planning and control (Dowd et al., 2012), and visuomotor coordination (Crippa et al., 2013). Gamified tasks presented on desktop computers could be easily completed by the participants after simple instructions or demonstrations provided by the test administrator (Aresti-Bartolome et al., 2015; Chaminade et al., 2015; Crippa et al., 2013; Deschamps et al., 2014; Dowd et al., 2012; Gardiner et al., 2017; Hetzroni et al., 2019; Li et al., 2016; Veenstra et al., 2012). Responses were provided using a variety of methods including taps on touch-sensitive screens (Chaminade et al., 2015; Crippa et al., 2013; Dowd et al., 2012; Gardiner et al., 2017; P. Li et al., 2016), mouse clicks (Hetzroni et al., 2019; Veenstra et al., 2012), pressing buttons on a button box or the keypad (Crippa et al., 2013), or using a stylus (Dowd et al., 2012). Data were automatically recorded and stored on the device.

Executive functioning (EF): Three of the nine studies assessed EF, one using a battery of established tasks (Gardiner et al., 2017), one using a novel set of tasks (Aresti-Bartolome et al., 2015), and one using a commercially available game (Veenstra et al., 2012). Primary metrics for the established EF tasks and the commercial game were accuracy (correct trials divided by the total number of trials), omission errors (no response when a response was required), and commission errors (response provided when no response was required). The commercial game also assessed reaction time, repeated number of clicks on the same object, and variability

in responses across trials. EF was also assessed using a multi-step planning game, an adaptation of the Tower of Hanoi, as part of the suite of established EF tasks. The primary metric was the number of moves in each correct trial (Gardiner et al., 2017). As seen in executive tasks presented on mobile devices, results related to accuracy were variable, with one study showing no group differences (Gardiner et al., 2017), the other showing reduced accuracy in the ASD group (Veenstra et al., 2012). The study assessing reaction time found the ASD group slower (Veenstra et al., 2012).

Metrics for the novel EF game were task completion (proportion of participants completing the game) and the number of pre-specified items identified per trial (Aresti-Bartolome et al., 2015). Consistent with observations of reduced task completion described above (Carlsson et al., 2018), the ASD group completed fewer trials (Aresti-Bartolome et al., 2015). They were also more prone to errors, although statistical significance was not determined (Aresti-Bartolome et al., 2015).

Cognitive: One study used a unique gamified task to assess abstract or relational modes of thinking (accuracy in correctly identifying the relationship between two objects as against the perceived form of the objects themselves) (Hetzroni et al., 2019). In this task, the correct response corresponded to the option where a different set of images are presented in the same spatial orientation as in the target image. In comparison to the TD group, the ASD group was compromised on identifying relationships between objects as they were more likely to select the option that contained components of the target image, with little attention to their spatial organization. It remains unclear whether this performance difference resulted from an impairment in relational thinking, a narrow and localized field of attention, or a differing interpretation of verbal instructions.

Social: The novel EF task (Aresti-Bartolome et al., 2015) also included a component wherein the game stopped randomly in the middle of the trial and the participant was required to interact with the test administrator to resume the game. The primary metrics were the latency to initiate an interaction, and whether eye contact was

made during the interaction. The ASD group took significantly longer to initiate the interaction, and were less likely to make eye contact with the test administrator (Aresti-Bartolome et al., 2015). Other studies assessed anthropomorphic bias (proportion of taps on videos with human characters exhibiting biological motion) (Chaminade et al., 2015) and prosocial behavior (proportion of responses to a distressed avatar) (Deschamps et al., 2014). The ASD group showed no preference for biological motion in human characters (Chaminade et al., 2015) as opposed to the TD comparison group. No group differences were reported for prosocial behavior (Deschamps et al., 2014).

Motor: Two studies used desktop computers to assess motor skills. One of them measured motor planning and control skills using a point-to-point movement task where the child was required to draw a line on the vertical plane using a stylus from a start position at the bottom of the screen to a target position at the top. Some trials included distractors near the target endpoint. A range of kinematic variables were estimated including the variability in movement preparation time across trials, and change in response metrics in the presence of a distractor (Dowd et al., 2012). The ASD group showed higher variability in latency (defined as movement preparation time in the study) compared to the TD group and did not adapt their movements in the presence of a distractor (Dowd et al., 2012). The second study assessed eye-hand coordination. The primary metric was Pearson's correlation between eye fixation latency on a target stimulus and reaction time of the hand response to indicate the left-right position of the stimulus on the screen, either using a button box, pressing pre-specified keys on the keypad, or touching the stimuli on the screen using a stick (Crippa et al., 2013). The ASD group demonstrated lower visuomotor coordination (Crippa et al., 2013).

Video recording of child behaviour

Facial expressions: Two studies from the same group (Borsos & Gyori, 2017; Gyori et al., 2018) analyzed facial expressions elicited by a deception and sabotage game to discriminate between groups. A webcam captured videos of the child which were then analyzed by the Noldus FaceReader. The first study exploring

differences in the intensity of various emotions averaged over different time intervals found no group differences (Gyori et al., 2018). However, a more granular analysis in the second study, exploring the mean and variance of emotion intensities frame-by-frame, found both the mean and variance of the 'scared' and 'surprised' expressions to be significantly higher in the ASD group compared to the TD group, as was their speed of change to a different expression (Borsos & Gyori, 2017). This result during active gameplay was in contrast to an earlier study using passive viewing of animated videos (Carpenter et al., 2021) which reported more neutral expressions in the ASD group. The second study (Borsos & Gyori, 2017) also assessed the ratio of valid to invalid frames, where invalid frames were defined as those in which the Noldus FaceReader was unable to identify the face, or unable to assign an emotion to the frame (Borsos & Gyori, 2017); no significant group differences were found.

Social: Two studies used computer vision analysis of head (Martin et al., 2018) and eye movements (Li et al., 2020) to discriminate between the TD and ASD groups. Videos were captured using webcams mounted on the monitor. In the first study (Martin et al., 2018), children were shown social and non-social videos on the desktop screen while the webcam captured their behaviours. Primary metrics were automated assessments of head movements (degrees of pitch, yaw and roll). The ASD group made greater lateral head movements, looking away from social videos. In the second study (Li et al., 2020), children viewed a picture of their mother on the screen. ML methods were used to compute the trajectories of their eye movements as captured by the webcam. The primary metric was the accuracy of a second ML algorithm to classify children into their diagnostic groups using features extracted from the length and angle information of children's eye movement trajectories. A classification accuracy of 92.6% was achieved, although it is not clear from the report whether this high accuracy was a result of over-fitting to a training set that also had been used for testing. Consistent with other studies (Bovery et al., 2018; Campbell et al., 2018), task engagement (proportion looking time at the screen) was found to be significantly lower in the ASD group in this study (Li et al., 2020), though no differences were reported in the former study (Martin et al., 2018).

Speech and Language

Two studies used picture stimuli presented on a desktop computer to assess speech characteristics (pitch) (Nakai et al., 2014) or acquired vocabulary and comprehension (Lin et al., 2013). A microphone attached to the child's clothing was used to record speech in the former study, which was then used to extract pitch characteristics using an ML algorithm. In the second study, correct or incorrect responses were recorded by key presses on the keyboard. The primary metrics were accuracy in naming and describing objects presented on the screen either in visual or audio format. In the first study, significant group differences were found in the variability of pitch metrics in older (7-9 years) but not in younger (4-6 years) children (Nakai et al., 2014). The second study found better language proficiency (vocabulary, comprehension, homographs and decoding) in the ASD group at younger ages (4-5 years) in most tasks, but the advantage decreased by the time children turned 6 years (Lin et al., 2013). The ASD group was also found to be more receptive to visual stimuli, as they were more accurate in articulating the names and descriptions of stimuli presented visually, as against stimuli presented in audio format (Lin et al., 2013). This visual bias was evident in that the auditory sentence comprehension task was the only one in which the TD group outperformed the ASD group.

Tasks using desktop technology were completed in 23 minutes on average (range = 10-40 mins) based on studies in which the completion time was reported, except for one study which took 90-120 minutes (Gardiner et al., 2017). This is ~3 times longer than the mean duration of tasks presented on portable devices (8 mins). The majority of these tasks (8/15; 53.3%) were administered in the laboratory (Borsos & Gyori, 2017; Chaminade et al., 2015; Crippa et al., 2013; Dowd et al., 2012; Gardiner et al., 2017; Gyori et al., 2018; Lin et al., 2013; Martin et al., 2018). Five studies reported the study setting as schools and daycares (Aresti-Bartolome N. et al., 2015; Peter K H Deschamps et al., 2014; Hetzroni et al., 2019; J. Li et al., 2020; Veenstra et al., 2012), and two studies (P. Li et al., 2016; Nakai et al., 2014) did not report the setting for data collection.

The majority of these tasks (8/15; 53.3%) were administered in the laboratory (Borsos & Gyori, 2017; Chaminade et al., 2015; Crippa et al., 2013; Dowd et al., 2012; Gardiner et al., 2017; Gyori et al., 2018; Lin et al., 2013; Martin et al., 2018). Five studies reported the study setting as schools and daycares (Aresti-Bartolome N. et al., 2015; Peter K H Deschamps et al., 2014; Hetzroni et al., 2019; J. Li et al., 2020; Veenstra et al., 2012), and two studies (P. Li et al., 2016; Nakai et al., 2014) did not report the setting for data collection.

2.2) Virtual reality (VR) platforms

Four studies (10.5%) used non-portable technology in the form of virtual reality platforms to assess joint attention (Jyoti & Lahiri, 2020; Shahab et al., 2018), motor imitation (Alcañiz Raya et al., 2020; Shahab et al., 2018) and visuomotor coordination (Jung et al., 2006). These studies used different VR platforms of varying levels of sophistication. The oldest (Jung et al., 2006) used a simple set of devices including a personal computer, projector, screen, infrared reflectors and a digital camera. On the other hand, one of the more recent studies (Alcañiz Raya et al., 2020) used the highly sophisticated CAVE-Automatic Virtual Environment (CAVE^{TM}) which includes a semi-immersive room with rear-projected surfaces. In this environment, the participant was not only able to see and hear an avatar, but also smell the food the avatar ate (Alcañiz Raya et al., 2020). The digital cameras used to record child responses included depth information.

Joint attention (JA): Two of the four studies assessed JA (Jyoti & Lahiri, 2020; Shahab et al., 2018) using a paradigm wherein an avatar directed their eye gaze towards virtual objects, and the child was expected to follow the gaze and provide a response, either by naming the object (Shahab et al., 2018), or by touching the target object on a touch-sensitive monitor (Jyoti & Lahiri, 2020). In the latter study, an avatar provided increasing numbers of cues towards the target object, first by gaze alone, followed up by both gaze and head-turn, then gaze, head-turn and finger-pointing, and finally, sparkling of the target in addition to all of the above cues (Jyoti & Lahiri, 2020). The primary metric was the number of times the target object was identified (Jyoti & Lahiri, 2020; Shahab et al., 2018). In both cases, the ASD

group scored lower than the TD group, especially when the cues were limited to gaze and head-turn alone, with performance improving as the number of cues provided increased. One of the studies recorded the reaction time (Jyoti & Lahiri, 2020) (latency between cue provided and target identification) and found the ASD group significantly slower than the TD group.

Motor imitation: Two studies assessed motor imitation (Alcañiz Raya et al., 2020; Shahab et al., 2018) using a VR set-up, one in which the child imitated virtual robots to play the drum and the xylophone (Shahab et al., 2018), and the other where they imitated various actions of an avatar appearing on the screen (Alcañiz Raya et al., 2020). Children were videotaped to record their responses. The primary metrics, respectively, were performance scores (correct imitations of robot or avatar actions) (Shahab et al., 2018), and the accuracy of an ML algorithm to classify children into their diagnostic groups using metrics calculated from the movements of joints (heads, limbs and trunk) across different types of actions (Alcañiz Raya et al., 2020). Both studies found the ASD group to be compromised in motor imitation. In the second study, the prediction accuracies of the ML methods were highest (89.36% with leave-one-out cross-validation) when using features from head movements alone as compared to using all available features. One of the studies assessed task engagement (defined as the duration for which the child played the game) (Shahab et al., 2018), and found the ASD group to be less engaged, also observed in several studies described above (Bovery et al., 2018; Campbell et al., 2018; J. Li et al., 2020).

Visuomotor coordination: One study used a VR platform to assess visuomotor coordination (Jung et al., 2006). The task involved popping virtual balloons with a real stick. The primary metrics were accuracy, reaction time and the total distance the stick was moved. While no group differences were observed in accuracy, reaction times in the ASD group were slower, as demonstrated by a few studies described above (Jones et al., 2018; Jyoti & Lahiri, 2020; Veenstra et al., 2012). A composite

principal-components measure based on the three primary metrics showed that the ASD group was less efficient (popped fewer balloons, took more time to pop each balloon, and moved the tangible stick more) in popping balloons in this task. Tasks took 14.6 min to complete on average (range = 10-20 min).

Supplementary Table 1: Search strategy

Keywords not included in the Phase 2 search are highlighted in red in the Phase 1 list

Domain	Participants	Digital Tool	Developmental domain	Disorder
**Keywords (Phase 1: May 2018)**	child* OR adolescen* OR student* OR pediatric* OR toddler* OR preschool* OR young OR infant	videogame OR gamif* OR game* OR "serious game"* OR gamelike* OR tablet* OR iPad OR computer* OR laptop* OR "virtual reality" OR Wii OR Xbox OR Nintendo OR console OR digital* OR web* OR PC OR phone* OR mobile* OR device OR tool OR "computer vision" OR "artificial intelligence" OR "machine learning" OR "deep learning"	cognit* OR brain OR memory OR attention OR reasoning OR visual-spatial OR visuo-spatial OR recall OR recognition OR "problem solving" OR "reaction time" OR vigilance OR "executive function" OR psycho* OR perception OR visu* OR inhibition OR "processing speed" OR motor OR lang* OR speech OR social* OR prosocial* OR emoti* OR adapti*	autis* OR ASD OR ADHD OR hyperactivity
**Keywords (Phase 2: Oct 2020)**	child* OR student* OR pediatric* OR toddler* OR preschool* OR young OR infant	Same as above	Same as above	autis* OR ASD

Supplementary Table 2: Additional participant details

Citation	Recruitment Setting	Demographic Details	Inclusion/ Exclusion criteria	Autism Diagnostic Criteria	# Recruited and reasons for loss of participants
Anzulewicz et al. 2016	ASD: Specialist therapeutic centres<br>TD: Regular kindergartens.	Not specified	**Inclusion**<br>- normal or corrected-to-normal vision<br>- no other sensory or motor deficits<br><br>**Exclusion**<br>- not able to follow simple instructions<br>- clinician or teacher uncertain about child's diagnosis or health	**Criteria:** ICD-10<br><br>**Personnel:** medical practitioners<br><br>**Functioning:** Full range of abilities	**Recruited:**<br>ASD = 37; TD = 45<br>**Loss:** ASD = 2<br>- 2 did not complete task
Ruta et al. 2017	ASD: Clinical facilities of National Research Council of Italy, Messina.<br>TD: Two mainstream nursery schools in Messina and Taormina (Sicily, Italy).	Not specified	Not specified	**Criteria:** DSM-5, ADOS<br><br>**Personnel:** multidisciplinary team (2 child psychiatrists, 2 developmental psychologists)<br><br>**Functioning:** Performance DQ (GMDS) > 85	**Recruited:**<br>ASD = 25; TD = 38<br>**Loss:** ASD = 4; TD = 1<br><br>- 5 did not pass pilot phase<br>- 1 (TD) due to GMDS sub-score not available<br>- 1 did not complete control task; excluded from relevant analysis.
Chetcuti et al. 2017	ASD: ASD-specific community support service or an established university research participant pool.<br>TD: Not specified	- Groups from similar ethnic backgrounds	**Exclusion**<br>- chronological age < 24 months.	**Criteria:** ADOS-2, ADOS-2 SA CSS (Autism Diagnostic Observation Schedule, Second Edition Social Affect Calibrated Severity Score)<br><br>**Personnel:** independent, research-reliable assessor<br><br>**Functioning:** Not specified	**Recruited:**<br>ASD = 35; TD = 20<br>**Loss:** None

Citation	Recruitment Setting	Demographic Details	Inclusion/ Exclusion criteria	Autism Diagnostic Criteria	# Recruited and reasons for loss of participants
Carlsson et al. 2018	ASD: Child Neuropsychiatry Clinic in Gothenburg, Sweden<br>TD: Three elementary schools in western Sweden.	Not specified	**Inclusion**<br>- age ≥ 5 years<br>- standard score ≥ 70 on Test for Reception of Grammar (v2)<br>- no reported ASD diagnosis (TD group)	**Criteria:** ADOS-G<br><br>**Personnel:** multi-disciplinary team (child and adolescent psychiatrist, neuropsychologist, speech-language pathologist)<br><br>**Functioning:** Full range of abilities	**Recruited:**<br>ASD = 71; TD = 98<br>**Loss:** ASD = 19<br>- 3 due to experimenter or technical errors<br>- 16 did not complete task
Jones et al. 2018	ASD: Center for Autism and the Developing Brain (CADB) and the Sackler Institute for Developmental Psychobiology.<br>TD: Not Specified	Not specified	**Inclusion**<br>TD:<br>- SCQ scores < 16<br>- SRS score < 70<br><br>ASD:<br>- diagnosed by research reliable clinician<br>- completed Autism Diagnostic Observation Schedule (ADOS) prior to participation.	**Criteria:** ADOS, clinical judgement<br><br>**Personnel:** research reliable clinicians<br><br>**Functioning:** Most children with IQ scores within standardized norms	**Recruited:**<br>ASD = 57; TD = 73<br>**Loss:** ASD = 1, TD<br>- cognitive scores not available<br>- 2 due to low developmental scores<br>- 2 due to poor behavioural performance<br>- 1 based on previous diagnosis of social pragmatic communication disorder
Campbell et al. 2019	ASD and TD: Primary care paediatric clinics and community advertisement.	- All English speaking participants<br>- No group differences in race (p = 0.56)	**Exclusion**<br>- known vision or hearing deficits<br>- did not hear English at home<br>- parents/ guardians did not speak and read English sufficiently to provide informed consent	**Criteria:** expert clinical judgment, ADOS-Toddler Module<br>M-CHAT-R/F to screen for ASD during recruitment<br><br>**Personnel:** licensed clinical psychologist with expertise in ASD	**Recruited:**<br>ASD = 22; TD = 85<br>**Loss:** TD = 3<br>- 1 did not complete task<br>- 2 due to incomplete data transfer

Citation	Recruitment Setting	Demographic Details	Inclusion/ Exclusion criteria	Autism Diagnostic Criteria	# Recruited and reasons for loss of participants
				**Functioning:** Mean (SD) MSEL Early Learning Composite score = 63.58 (25.95)	
Gale et al. 2019	ASD: Treatment centres<br>TD: Nursery (the majority) or via acquaintances of first author	Not specified	**Inclusion**<br>ASD:<br>- developmental age: ≤ 5 yrs<br>TD:<br>- no psychiatric diagnosis<br>- no concerns about child's development raised by parents or professionals<br>- chronological age: ≤ 5 yrs<br>Both:<br>- no medical conditions that can interfere with study (uncontrolled epilepsy, major motor or sensory impairments)	**Criteria:** ICD-10, CARS-2 (ASD group only)<br><br>**Personnel:** medical professional independent of the study<br><br>**Functioning:** Developmental age (BSID-III) matched with TD chronological age.	**Recruited:**<br>Study 1: ASD = 27; TD = 40<br>Study 2: ASD = 19; TD = 21<br>Study 3: ASD = 17; TD = 23<br>**Loss:** None
Carpenter et al. 2020	ASD and TD: Primary care paediatric clinics and community advertisement.	- All English speaking participants<br>- No group differences in race (p = 0.56)	**Exclusion**<br>- known vision or hearing deficits<br>- did not hear English at home<br>- parents/ guardians did not speak and read English sufficiently to provide informed consent	**Criteria:** expert clinical judgment, ADOS-Toddler Module M-CHAT-R/F to screen for ASD during recruitment<br><br>**Personnel:** Licensed clinical psychologist with expertise in ASD<br><br>**Functioning:** Mean (SD) MSEL Early Learning Composite score = 63.58 (25.95)	**Recruited:**<br>ASD = 22; TD = 75; DD = 8<br>**Loss:** TD = 1<br>- 1 did not complete task
Zhao et al. 2020	ASD: Guangzhou Children's Care Centre<br>TD: Amy Education School in Zhengzhou	Not specified	**Inclusion**<br>TD: No Autistic history<br>ASD: ASD diagnosed by specialists	Not specified<br><br>**Functioning:** Not specified	**Recruited:**<br>ASD = 10; TD = 10<br>**Loss:** None

Citation	Recruitment Setting	Demographic Details	Inclusion/ Exclusion criteria	Autism Diagnostic Criteria	# Recruited and reasons for loss of participants
Bovery et al. 2021	ASD and TD: Primary care paediatric clinics and community advertisement. Participants approached during 18- or 24-month well child visit in paediatric clinics	- All English speaking participants<br>- No group differences in race (p = 0.56)	**Exclusion**<br>- known vision or hearing deficits<br>- did not hear English at home<br>- parents/ guardians did not speak and read English sufficiently to provide informed consent	**Criteria:** Expert clinical judgement, ADOS-TF<br><br>M-CHAT-R/F to screen for ASD during recruitment<br>**Personnel:** licensed clinical psychologist with expertise in ASD<br><br>**Functioning:** Mean (SD) MSEL Early Learning Composite score = 63.58 (25.95)	**Recruited:**<br>ASD = 22; TD = 85<br>**Loss:** TD = 3<br>- 1 did not complete task<br>- 2 due to incomplete data transfer<br>(Based on information in Campbell et al. 2018)
Lu et al. 2019	Not specified	Not specified	Not specified	**Criteria:** DSM-IV-TR<br><br>**Personnel:** paediatricians<br><br>**Functioning:** Matched with TD group on non-verbal IQ (Combined Raven's test) and verbal mental ability (PPVT-R)	**Recruited:**<br>ASD = 28; TD = 28<br>**Loss:** None
Nakai et al. 2014	ASD: Kobe University Hospital Developmental Behavioural Paediatric Clinic<br>TD: Mainstream preschool or primary schools in regions where children with ASD resided	Not specified	**Inclusion**<br>ASD:<br>- no obvious neurological symptoms or comorbid disorder<br>- able to understand simple instructions and express ≥ 30 words<br>- diagnosed with ASD (DSM-5 criteria)<br><br>TD:<br>- no history of special education<br>- no speech, communication, or learning problems	**Criteria:** DSM-IV-TR<br><br>**Personnel:** expert child neurologist<br><br>**Functioning:** Mean IQ score (Tool unknown) = 69.31	**Recruited:**<br>ASD = 26; TD = 37<br>**Loss:** None
Wijesinghe et al. 2019	ASD and TD: Lady Ridgeway Hospital for children, Colombo, Sri Lanka	Not specified	Not specified	Not specified<br><br>**Functioning:** Not specified	**Recruited:**<br>ASD = 8; TD = 9<br>**Loss:** None

Citation	Recruitment Setting	Demographic Details	Inclusion/ Exclusion criteria	Autism Diagnostic Criteria	# Recruited and reasons for loss of participants
Gyori et al. 2008	Not specified	Not specified	Not specified	**Criteria:** ADOS, ADI-R<br><br>**Personnel:** Not specified<br><br>**Functioning:** Matched with TD group on IQ (Leiter-R)	**Recruited:**<br>ASD = 13; TD = 13<br>**Loss:** None
Lin et al. 2013	ASD: Chang Gung Memorial Hospital, Tao-Yuan, Taiwan<br>TD: 4 geographic areas in Tao-Yuan County, Taiwan.	Not specified	Not Specified	**Criteria:** Not specified<br><br>**Personnel:** pediatric psychiatrists<br><br>**Functioning:** Not specified	**Recruited:**<br>ASD = 35; TD = 300<br>**Loss:** None
Chaminade et al. 2013	Not specified	Not specified	Not specified	**Criteria:** DSM-IV, ADOS<br><br>**Personnel:** Not specified<br><br>**Functioning:** Developmental age matched with TD chronological age<br>- Mean (SD) mental ability = 35 (8) months (PEP3 - Revised)	**Recruited:**<br>ASD = 12; TD = 24<br>**Loss:** None
Deschamps et al. 2014	ASD: Department of Child and Adolescent Psychiatry (outpatient department), University Medical Center, Utrecht.<br>TD: Regular elementary schools in Utrecht.	Not specified	**Inclusion**<br>TD:<br>- no history of clinical diagnosis of ASD<br>- Total SRS score < 60<br>- IQ > 70.	**Criteria:** DSM-IV<br><br>**Personnel:** child and adolescent psychiatrist<br><br>**Functioning:** Matched with TD group on IQ (WISC-III Dutch version)	**Recruited:**<br>ASD = 27; TD = 29<br>**Loss:** ASD = 5<br>- 2 did not have clinical diagnosis of ASD<br>- 3 had IQ < 70
Aresti-Bartolome et al. 2015	Not specified	Not specified	Not Specified	**Criteria:** DSM (version not specified)<br><br>**Personnel:** professionals<br><br>**Functioning:** Performance DQ (GMDS) > 85	**Recruited:**<br>ASD = 20; TD = 20<br>**Loss:** None
Li et al. 2016	ASD: Special school for children with ASD.	Not specified	Not specified	**Criteria:** DSM-IV-TR confirmed by Chinese version of Autism Spectrum Quotient:	**Recruited:**

Citation	Recruitment Setting	Demographic Details	Inclusion/ Exclusion criteria	Autism Diagnostic Criteria	# Recruited and reasons for loss of participants
	TD: Regular school in Qingdao, China			Children's version (AQ-Child), Social Responsive Scale (SRS), and Social Communication Questionnaire (SCQ)<br><br>**Personnel:** professional clinicians<br><br>**Functioning:** Matched with TD group on Non-verbal IQ and Verbal mental age	ASD = 30; TD = 30<br>**Loss:** None
Borsos et al. 2017	Not specified	Not specified	**Exclusion**<br>- developmental disorders<br>- visual or motor impairments<br>- difficulties with using a computer mouse	**Criteria:** ADOS, ADI-R<br><br>**Personnel:** Not specified<br><br>**Functioning:** Matched with TD group on IQ (Leiter-R Brief)	**Recruited:** ASD = 13; TD = 13<br>**Loss:** None
Martin et al. 2018	ASD: Older siblings of infants recruited for longitudinal study of high-risk development.<br>TD: Community contact and older siblings of infants recruited for longitudinal study of high-risk development.	Not specified	**Inclusion**<br>- no reported risks or diagnoses at the time of study (TD group).<br><br>**Exclusion**<br>- gestational age < 37 weeks or major birth complications	**Criteria:** DSM-IV, ADOS, ADI-R<br><br>**Personnel:** Licensed psychologist unfamiliar with the child's previous diagnosis<br>**Functioning:** Matched with TD group on IQ (WPPSI or MSEL)	**Recruited:** ASD = 21; TD = 21<br>**Loss:** None
Li et al. 2019	ASD and TD: Primary and special education schools	Not specified	Not specified	**Criteria:** DSM-IV<br><br>**Personnel:** paediatric psychiatrists<br><br>**Functioning:** Matched with TD group on verbal mental age (PPVT-R)	**Recruited:** ASD = 136; TD =136<br>**Loss:** None
Li et al. 2019	ASD: Training centre for children with special needs in Shenzhen<br>TD: Kindergarten and primary school in Shenzhen	Not specified	**Inclusion**<br>- no reported language, hearing or cognitive deficits	**Criteria:** Chinese Classification of Mental Disorders Version 3 (CCMD-3) (Chinese Society of Psychiatry, 2001) based on DSM-IV and ICD-10	**Recruited:** ASD = 17; TD = 17<br>**Loss:** None

Citation	Recruitment Setting	Demographic Details	Inclusion/ Exclusion criteria	Autism Diagnostic Criteria	# Recruited and reasons for loss of participants
				**Personnel:** specialists or psychiatrists<br><br>**Functioning:** Not specified	
Shahab et al. 2017	Not specified	Not specified	Not specified	Not specified<br><br>**Functioning:** Not specified	**Recruited:** ASD = 14; TD = 21<br>**Loss:** None
Jyoti et al. 2020	ASD: Mental health institute<br>TD: Neighbouring regular school	Not specified	**Inclusion**<br>- comfortable with using touch screen on phones	**Criteria:** Social Responsiveness Scale (SRS; score ≥ 59) and Social Communication Questionnaire (SCQ; score ≥ 15)<br><br>**Personnel:** Not specified<br><br>**Functioning:** Not specified	**Recruited:** ASD = 20; TD = 20<br>**Loss:** None
Moradi et al. 2017	ASD: Center for the Treatment of Autistic Disorders (CTAD), Tehran<br>TD: Kindergarten located near CTAD	Not specified	**Inclusion**<br>TD: no developmental or mental disorders	**Criteria:** DSM-IV, GARS, ADI-R<br><br>**Personnel:** two independent experts<br><br>**Functioning:** Not specified	**Recruited:** ASD = 25; TD = 25<br>**Loss:** ASD = 6, TD = 7<br>- short test time<br>- interruptions during the test<br>- unreliable recorded data
Rafique et al. 2019	ASD: Autism Learning Institutes<br>TD: Regular kindergartens	Not specified	**Inclusion**<br>TD: no symptoms of ASD<br><br>ASD: Medically diagnosed ASD	Not specified<br><br>**Functioning:** Not specified	**Recruited:** ASD = 22; TD = 22<br>**Loss:** None
Mahmoudi-Nejad et al. 2017	Not specified	Not specified	Not specified	Not specified<br><br>**Functioning:** Not specified	**Recruited:** ASD = 5; TD = 7<br>**Loss:** None
Dawson et al. 2018	ASD and TD: Primary care paediatric clinics, referral from physicians, and community advertisement.	- Ethnic/racial composition of ASD and TD groups comparable.	**Exclusion**<br>- known vision or hearing deficits<br>- do not hear English at home<br>- caregivers do not speak and read	**Criteria:** expert clinical judgment, ADOS<br>M-CHAT-R/F for screening during recruitment	**Recruited:** ASD = 22; TD = 82<br>**Loss:** None

Citation	Recruitment Setting	Demographic Details	Inclusion/ Exclusion criteria	Autism Diagnostic Criteria	# Recruited and reasons for loss of participants
			English sufficiently to provide informed consent	**Personnel:** Licensed clinical psychologist with expertise in ASD<br><br>**Functioning:** Mean (SD) MSEL ELC score = 63.58 (25.95)	
Fleury et al. 2013	Not specified	Not specified	**Inclusion**<br>- normal or corrected-to-normal vision<br>- not using any medication which may affect motor function<br><br>**Exclusion**<br>- diagnosed with a genetic or metabolic disorder associated with autism	**Criteria:** DSM-IV, ADI-R, ADOS<br><br>**Personnel**: Not specified<br><br>**Functioning:** IQ scores > 70 (Stanford–Binet Intelligence Scales - 5th edition)	**Recruited:** ASD = 23; TD = 20<br>**Loss:** ASD = 8, TD = 1<br>- 6 failed to complete task<br>- 3 (ASD) had FSIQ < 70
Dowd et al. 2012	ASD: Autism Victoria and other early intervention and social playgroups.<br>TD: Not specified	Not specified	**Exclusion**<br>ASD: Comorbid seizure, neurological, or genetic condition	**Criteria:** DSM-IV-TR, ADOS (5 children)<br><br>**Personnel:** professional with expertise in autism not associated with project<br><br>**Functioning:** Matched with TD group on performance IQ (WISC-R-IV or WPPSY-III)	**Recruited:** ASD = 13; TD = 13<br>**Loss:** ASD = 2, TD = 1<br>- 3 due to poor compliance and inability to complete task
Crippa et al. 2013	ASD: Author institute<br>TD: Through local paediatricians.	Not specified	**Inclusion**<br>- normal or corrected to normal vision<br>- drug-naive<br>- full scale IQ score > 70 (WPPSI or WISC-R)<br><br>**Exclusion**<br>TD group:<br>- suspected signs of social/communicative disorders<br>- developmental abnormalities	**Criteria:** DSM-IV TR<br><br>**Personnel:** medical doctor specialized in child neuropsychiatry with expertise in autism. Confirmed independently by child psychologist (clinical judgement through observation and discussion with parent)<br><br>**Functioning:** Matched with TD group on IQ (WISC-R and WPPSI)	**Recruited:** ASD = 14; TD = 14<br>**Loss:** None

Citation	Recruitment Setting	Demographic Details	Inclusion/ Exclusion criteria	Autism Diagnostic Criteria	# Recruited and reasons for loss of participants
			- medical disorders with central nervous system implications		
Jung et al. 2006	ASD: Children's Hospital in Seoul (Outpatient unit).<br>TD: Kindergarten belonging to a University in Seoul	Not specified	Not Specified	**Criteria:** DSM-IV<br><br>**Personnel:** Not specified<br><br>**Functioning:** Mean IQ score (Tool unknown) = 64	**Recruited:**<br>ASD = 12; TD = 20<br>**Loss:** None
Raya et al. 2020	ASD: Development Neurocognitive Centre, Red Cenit, Valencia, Spain.<br>TD: Recruited by management company through mailings to families.	Not specified	Not specified	**Criteria:** ADOS-2, ADI-R<br><br>**Personnel:** Not specified<br><br>**Functioning:** Not specified	**Recruited:**<br>ASD = 24; TD = 25<br>**Loss:** None
Chen et al. 2017	ASD: Special school<br>TD: Regular kindergartens	Not specified	**Inclusion (both)**<br>- normal or corrected visual acuity<br>- no other sensory or motor deficits.<br><br>ASD:<br>- free of medication, history of traumatic brain injury or other neurological illnesses.<br><br>TD:<br>- no disease, intellectual disability, learning disability, or other developmental obstacles certified by a clinician<br><br>**Exclusion**<br>- intellectual disability<br>- unable to follow simple instructions	**Criteria:** DSM-5<br><br>**Personnel:** psychologists or clinicians<br><br>**Functioning:** Children with learning disabilities or ID were excluded.	**Recruited:**<br>ASD = 40; TD = 51<br>**Loss:** None
Hetzroni et al. 2019	ASD, TD and NDD: Schools	- All children spoke Hebrew as their first language	Not specified	**Criteria:** DSM-5, CARS-2-HF (score ≥ 27.5)<br><br>**Personnel:** Not specified	**Recruited:**<br>ASD = 24; TD = 24; IDD = 24<br>**Loss:** None

Citation	Recruitment Setting	Demographic Details	Inclusion/ Exclusion criteria	Autism Diagnostic Criteria	# Recruited and reasons for loss of participants
				**Functioning:** Matched with TD group on verbal IQ (PLS-4), non-verbal IQ and receptive vocabulary (WPPSI-III)	
Veenstra et al. 2012	ASD: Four medical day care centres in the Netherlands<br>TD: General population sample	- Lower-educated parents in ASD group compared to TD group	**Inclusion**<br>TD: Rated as effective learners by three raters (trained rater, parents, teachers)	**Criteria:** DSM-IV<br><br>**Personnel:** licensed psychologist<br><br>**Functioning:** Matched with TD group on IQ (MSEL)	**Recruited:**<br>ASD = 13; TD = 5<br>**Loss:** None
Gardiner et al. 2017	Not specified	- Maternal education ranging from less than high school to graduate degree.<br>- Groups from similar ethnic backgrounds	**Inclusion**<br>TD:<br>- no history of learning disabilities, neurological disorders, or psychiatric conditions<br>- IQ > 70 (both groups)	**Criteria:** DSM-IV-TR, ADI-R, ADOS. ASRS as a measure of symptom severity<br><br>**Functioning:** Matched with TD group on NVIQ (Stanford-Binet Intelligence Scales)<br><br>**Personnel:** qualified paediatrician, registered doctoral-level psychologist, or psychiatrist	**Recruited:**<br>ASD = 32; TD = 22<br>**Loss:** ASD = 8, TD = 3<br>- 10 had IQ < 70.<br>- 1 undiagnosed TD child suspected of having ASD

Supplementary Table 3: List of questions to assess risk of bias

SR #	Question
1	Were the aims and objectives sufficiently described?
2	Were the groups comparable other than the presence of disease in cases or the absence of disease in controls?
3	Were cases and controls matched appropriately?
4	Was the design appropriate to measure specificity of ASD symptoms?
5	Were the same criteria used for identification of cases and controls?
6	Was exposure measured in a standard, valid and reliable way for both cases and controls?
7	Were outcomes assessed in a standard, valid and reliable way for both cases and controls?
8	Was appropriate statistical analysis used?
9	Was the estimate of variance reported for the main results?
10	Were the results were sufficiently described?
11	Did the results support the conclusions?
12	Were the limitations of the study discussed?
13	Were the reasons for loss of participants described?

Supplementary Figure 1: Examples of digital tools used for identifying risk of ASD during early childhood.

Please refer to Table 1 for details of tool implementation

Citation	Type of tool	Image of tool / platform
Anzulewicz et al. 2016	**Gamified task**<br><br>Child plays two tablet based games - A: ‘Sharing’; B: ‘Creativity’<br><br>**License:**<br>https://s100.copyright.com/AppDispatchServlet?title=Toward%20the%20Autism%20Motor%20Signature%3A%20Gesture%20patterns%20during%20smart%20tablet%20gameplay%20identify%20children%20with%20autism&author=Anna%20Anzulewicz%20et%20al&contentID=10.1038%2Fsrep31107&copyright=The%20Author%28s%29&publication=2045-2322&publicationDate=2016-08-24&publisherName=SpringerNature&orderBeanReset=true&oa=CC%20BY	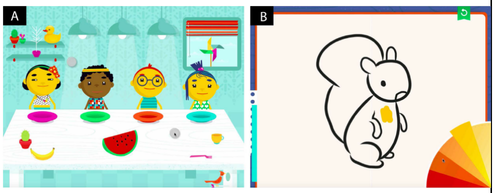
Ruta et al. 2017	**Gamified task**<br><br>Child presses one of two buttons to reveal the social or non-social stimulus as per their preference.<br><br>**License:**<br>https://s100.copyright.com/AppDispatchServlet?title=Reduced%20preference%20for%20social%20rewards%20in%20a%20novel%20tablet%20based%20task%20in%20young%20children%20with%20Autism%20Spectrum%20Disorders&author=Liliana%20Ruta%20et%20al&contentID=10.1038%2Fs41598-017-03615-x&copyright=The%20Author%28s%29&publication=2045-2322&publicationDate=2017-06-12&publisherName=SpringerNature&orderBeanReset=true&oa=CC%20BY	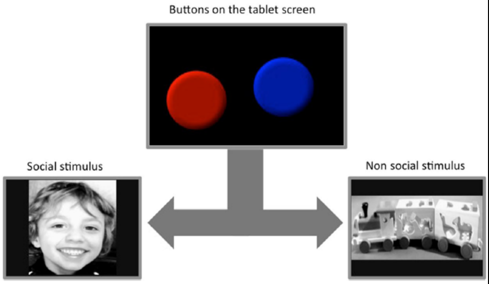
Carlsson et al. 2018	**Gamified task**<br><br>False Belief task with “Johanna” and “Jansson the Cat”. Child answers questions (Where will Johanna look for the ball? and Where is the ball?”) by pointing at one of the yellow circles on the touch screen<br><br>**License:**<br>https://creativecommons.org/licenses/by/4.0/	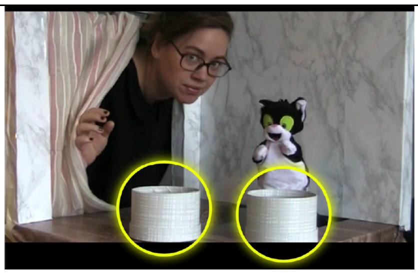
Gale et al. 2019	**Gamified task**<br><br>Child taps on one of two blurred images. When tapped, image becomes clearly visible for 2s. Different social and non-social images are presented in each trial.<br><br>**License:**<br>https://creativecommons.org/licenses/by/4.0/	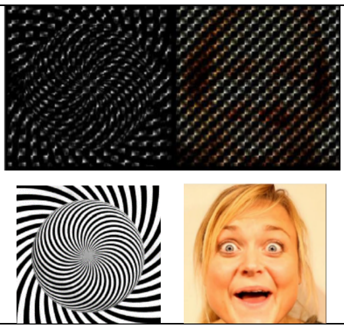
Nakai et al. 2014	**Analysis of speech characteristics**	No example image provided

Aresti-Bartolome et al. 2015	**Gamified task**<br><br>Child collects as many items as possible (target image demonstrated in right hand corner of the screen: red star in level 1). When the game stops, child has to interact with the administrator to restart game.<br><br>**License:**<br>This article is published with Open Access and distributed under the terms of the Creative Commons Attribution and Non-Commercial License	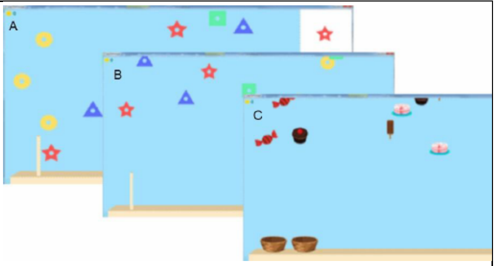
Martin et al. 2018	**Passive video recording of child behaviour (head movements)**<br><br>Social and non-social images (designed to elicit joint attention and emotion expression) presented on a video-monitor. Child video-recorded while watching these videos.<br><br>**License:**<br>https://s100.copyright.com/AppDispatchServlet?title=Objective%20measurement%20of%20head%20movement%20differences%20in%20children%20with%20and%20without%20autism%20spectrum%20disorder&author=Katherine%20B.%20Martin%20et%20al&contentID=10.1186%2Fs13229-018-0198-4&copyright=The%20Author%28s%29.&publication=2040-2392&publicationDate=2018-02-27&publisherName=SpringerNature&orderBeanReset=true&oa=CC%20BY%20%2B%20CC0	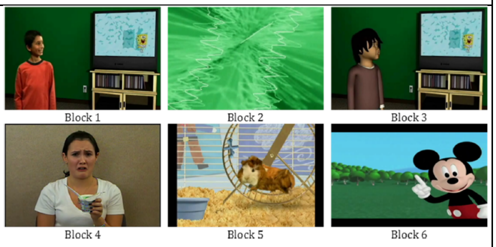
Raya et al. 2020	**Virtual reality platform**<br><br>Child to imitate avatars in a VR set-up while their movements are video-recorded.<br><br>**License:**<br>https://creativecommons.org/licenses/by/4.0/	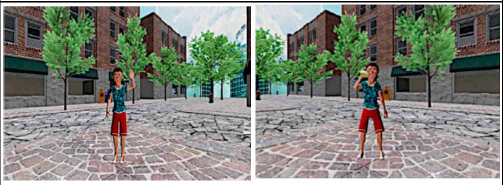

Supplementary Figure 2: Number of articles retrieved as a function of year

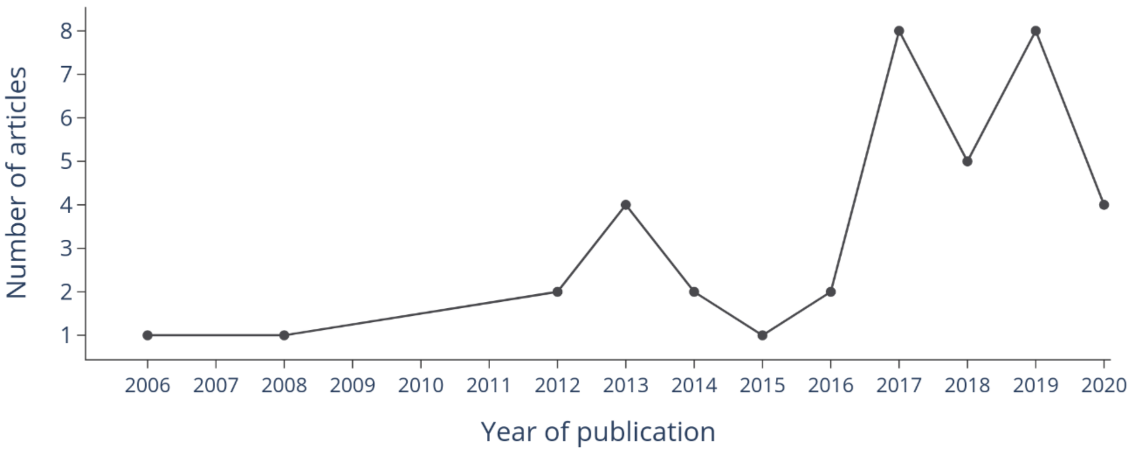

Supplement: sj-pdf-1-aut-10.1177_13623613221133176 – Supplemental material for Digital tools for direct assessment of autism risk during early childhood: A systematic review [file sj-pdf-1-aut-10.1177_13623613221133176.pdf]
